# Hierarchical Controlled Joint Remote Implementation of the Partially Unknown Operations of m Qudits via m High-Dimensional Entangled States

**DOI:** 10.3390/e26100857

**Published:** 2024-10-10

**Authors:** Ruiheng Jing, Qi Lan, Ping Zhou

**Affiliations:** 1 College of Mathematics and Physics, Guangxi Minzu University, Nanning 530006, China; bnuerr@tom.com (R.J.); pinggogo@tom.com (Q.L.); 2 Key Lab of Quantum Information and Quantum Optics, Guangxi University for Nationalities, Nanning 530006, China; 3 Guangxi Key Laboratory of Hybrid Computational and IC Design Analysis, Nanning 530006, China

**Keywords:** hierarchial joint remote implementation of quantum operation, partially unknown operation, high-dimensional quantum system

## Abstract

We present a protocol for the hierarchical controlled joint remote implementation of the partially unknown operations of m qudits belonging to some restricted sets by using m multiparticle high-dimensional entangled states as the quantum channel. All the senders share the information of the partially unknown operations and cooperate with each other to implement the partially unknown operations on the remote receiver’s quantum system. The receivers are hierarchized in accordance with their abilities to reconstruct the desired state. The agents in the upper grade need only cooperate with one of the lower-grade agents, and the agents in the lower grade need the cooperation of all the other agents. The protocol has the advantage of having high channel capacity by using a high-dimensional entangle state as the quantum channel for the hierarchial controlled joint remote implementation of partially unknown quantum operations of m qudits.

## 1. Introduction

The utilization of the principle of quantum mechanics in information processing provides some novel methods for quantum information processing, such as quantum key distribution [1,2,3,4,5,6,7,8,9], quantum secure direction communication [10,11,12,13,14,15,16,17,18,19,20], quantum teleportation [21,22,23,24,25,26,27,28], quantum remote state preparation [29,30], quantum computation [31,32,33,34,35,36,37,38,39,40], quantum nonlocal gate [41,42,43,44,45,46] and quantum operation remote implementation [47,48,49].

Recently, the remote implementation of quantum operation has garnered much interest since it was first proposed by Huelga et al. [47]. Theoretical protocols for the remote implementation of quantum operations, especially partially unknown quantum operations, have been proposed via different quantum channels [48,49,50,51,52,53,54,55,56,57,58,59,60,61]. The operations are partially unknown, since the values of their matrix elements are unknown but the positions of the nonzero matrix elements are known. Huelga et al. showed that single-qubit operations can be remote implemented via a quantum entangled channel shared in advance, and classical communication and partially unknown operations of one qubit belonging to two restricted sets

(1) $$\begin{array}{c} U_{com}=\left(\begin{array}{cc} e^{i\varphi } & 0\\ 0 & e^{-i\varphi } \end{array}\right),U_{anti}=\left(\begin{array}{cc} 0 & e^{i\varphi }\\ -e^{-i\varphi } & 0 \end{array}\right) \end{array}$$

can be remote implemented via less resources [48]. In 2006, Wang investigated the extension of the remote implementation of partially unknown operations to the case of multiqubit. The partially unknown operations of N qubits as suggested by Wang have only one nonzero element in every row or every column of their representation matrices. Since the nonzero element in the first row has $2^{N}$ possible positions, the nonzero element in the second row has $2^{N}-1$ possible positions and the nonzero element in the $2^{N}$th row has one possible position, the partial operations of N qubits belong to $2^{N}!$ restricted sets [49]. Moreover, Wang presented a scheme for combining the remote implementation of $U=U_{1}U_{2}$. $U_{1},U_{2}$ are partially unknown operations belonging to the restricted sets [50]. In 2008, Fan and Liu presented a protocol for the multiparty controlled remote implementation of partially unknown operations [51]. Qiu and Wang presented a scheme to implement the partially unknown operations of two qubits belonging to 24 restricted sets via Cavity QED [52]. In 2010, Chen showed that quantum operations belonging to restrict set can be divided into m pieces and simultaneously remotely implemented on m remote receivers’ quantum system [53]. In 2011, Chen et al. presented a protocol for the probabilistic remote implementation of a partially unknown operation via nonmaximally entangled state [54]. Situ and Qiu considered the remote implementation of partially unknown operations of multiqubit without prior sharing of entanglement [55]. In 2013, Zhan et al. presented a protocol for the remote implementation of partially unknown operations

(2) $$\begin{array}{c} U_{0}=\left(\begin{array}{ccc} u_{0} & 0 & 0\\ 0 & u_{1} & 0\\ 0 & 0 & u_{2} \end{array}\right),U_{1}=\left(\begin{array}{ccc} 0 & 0 & u_{0}\\ u_{1} & 0 & 0\\ 0 & u_{2} & 0 \end{array}\right),U_{2}=\left(\begin{array}{ccc} 0 & u_{0} & 0\\ 0 & 0 & u_{1}\\ u_{2} & 0 & 0 \end{array}\right) \end{array}$$

belonging to three restricted sets in a three-dimensional quantum system [56]. In 2019, Peng et al. put forward a protocol for quantum rotation operation sharing with a five-qubit cluster state [57]. In 2022, An and Cao presented a method for the parallel remote implementation of partially unknown operations of one qubit in polarization and spatial-mode degrees of freedom with a hyperentangled state [58]. In 2023, Peng et al. presented a scheme for the remote implementation of m partially unknown operations of one qubit on the remote receivers’ quantum systems under the controller’s control [59]. In 2024, Liu et al. proposed a protocol for the bidirectional controlled remote implementation of a partially unknown operation of two qubits belonging to eight restricted sets via a nine-qubit entangled state $\frac{1}{\sqrt{2}}[|\varphi^{+}\rangle |\varphi^{+}\rangle |\varphi^{+}\rangle |\varphi^{+}\rangle |0\rangle +|\varphi^{-}\rangle |\varphi^{-}\rangle |\varphi^{-}\rangle |\varphi^{-}\rangle |1\rangle ]$, where $|\varphi^{\pm }\rangle =\frac{1}{\sqrt{2}}(|00\rangle \pm \left|11\rangle \right)$ [60]. Shi et al. presented a protocol for the hierarchical joint remote implementation of a partially unknown quantum operation of one qubit with a cluster state, where the receivers are hierarchized according to their abilities to accomplish the remote implementation of the partially unknown operations [61]. The remote implementation of the partially unknown operation of one qubit has been experimental demonstrated via linear optical elements [62].

In the past few years, researchers have expressed much interest in quantum information processing via high-dimensional quantum system, as a high-dimensional quantum system has a high capacity for the storing and processing of quantum information in long-distance quantum communication. Moreover, it offers a alternate method for scaling up the quantum computation. In 2000, Muthukrishnan and Stroud showed that an arbitrary n-qudit operation can be decomposed into single- and two-qudit operations [63]. In 2001, Bennett investigated the method for the remote preparation of an arbitrary qudit state [64]. In 2002, Vlasov showed that two single-qudit noncommutative operations and two-qudit operations can construct a universal qudit operation [65]. In 2003, Zhou et al. presented the concept of a qudit cluster state and proposed one-way computation based on the qudit cluster state [66]. In 2005, Wang et al. presented a protocol for quantum secure direct communication via high-dimensional quantum state [67]. In 2007, Li et al. put forward a method for the controlled teleportation of an arbitrary m-qudit state with d-dimensional Greenberger–Horne–Zeilinger(GHZ) states [68]. In 2014, Luo and Wang proposed a protocol for the implementation of universal quantum computation on the high-dimensional quantum system via a set of one-qudit and two-qudit operations [69]. Krenn et al. proposed the creation of a (100×100)-dimensional entangled state via spatial modes of photons [70]. In 2017, Kues et al. demonstrated the generation of a high-dimensional frequency entangled state [71]. Bouchard et al. realized optimal cloning for a high-dimensional state of photons in their orbital angular momentum degrees of freedom [72]. In 2018, Hu et al. reported the experimental demonstration of quantum superdense coding with a four-dimensional path-polarization entangled state $|\psi \rangle =\frac{1}{2}(|00\rangle +|11\rangle +|22\rangle +\left|33\rangle \right)$ [73]. In 2019, Reimer et al. demonstrated high-dimensional one-way quantum computation via qudit cluster state [74]. In 2020, Vagniluca et al. realized four-dimensional quantum key distribution via high-dimensional quantum state encoded in time-bin degrees of freedom [75]. Hu et al. experimentally realized the efficient generation [76] and distribution [77] of a high-dimensional entangled state and demonstrated high-dimensional quantum teleportation via the high-dimensional entangled state [78]. Wang et al. investigated the control effectiveness of high-dimensional controlled teleportation [79]. Kiktenko et al. showed the significant reduction of quantum operations for the implementation of a Toffoli gate via a high-dimensional quantum state [80]. In 2022, Saha et al. presented a novel method to decompose an n-qudit Toffoli gate into two-qudit gates without an auxiliary qudit [81]. Nikolaeva proposed a scheme to decompose an n-qubit Toffoli gate via 2n-3 two-qutrit gates [82]. Chen et al. presented a scheme for the perfect teleportation of a sing-qubit state with a high-dimensional partially entangled state $|\Phi \rangle =a_{0}|00\rangle +a_{1}|11\rangle +a_{2}|22\rangle$ [83]. In 2023, Hrmo et al. experimentally realized a two-qudit entangling gate via a trapped-ion system [84]. Luo et al. experimentally demonstrated a two-qutrit gate via superconducting quantum circuits [85]. Xing proposed a method for preparing a multiparticle high-dimensional GHZ state via optical system [86]. In 2024, Lv et al. experimentally demonstrated high-dimensional controlled teleportation via a three-dimensional GHZ state $|\phi \rangle =\frac{1}{\sqrt{2}}(|000\rangle +|111\rangle +\left|222\rangle \right)$ [87]. Xu et al. experimentally demonstrated quantum state compression from two qubits α|0〉+β|1〉 to a qutrit $\alpha^{2}|0\rangle +\sqrt{2}\alpha \beta |1\rangle +\beta^{2}|2\rangle$ [88].

Although there are some protocols for the remote implementation of partially unknown operations belonging to restricted sets in a high-dimensional quantum system, the hierarchical joint remote implementation of partially unknown operations of m qudits in high-dimensional quantum system is not seriously considered [55,56]. We present a protocol for the hierarchical joint remote implementation of partially unknown operations of m qudits belonging to restricted sets via m multiparticle high-dimensional entangled states. All the senders share the information of the partially unknown operations and cooperate with each other to jointly remotely implement the partially unknown operations in high-dimensional system. The receivers are hierarchized in accordance with their abilities to complement the partially unknown operations remote implementation. The upper-grade agents only need the cooperation of one of the other agents to complete the remote implementation of the partially unknown operations and the lower-grade agents need the cooperation of all the other agents. The protocol has the advantage of having a high channel capacity by remote implementing partially unknown operations of m qudits via m multiparticle high-dimensional entangled states.

## 2. Hierarchial Joint Remote Implementation of Partially Unknown Operations of One Qudit via a Multiparticle High-Dimensional Entangled State

To present the principle of our protocol clearly, we first present the protocol for the hierarchical joint remote implementation of partially unknown operations of one qudit belonging to restricted sets in d-dimensional quantum system, then generalize it to the case of remote implementation of partially unknown operations of m qudits.

Similar to the two-dimensional system, |0〉,⋯,|d−1〉 is the eigenbasis of the pauli operator $Z_{d}$ [68,89]. ${|0\rangle }_{x},\cdots ,{|d-1\rangle }_{x}$ is the eigenbasis of the pauli operator $X_{d}$.

(3) $$\begin{array}{c} {|j\rangle }_{x}=\frac{1}{\sqrt{d}}(|0\rangle +e^{\frac{2\pi i}{d}j}|1\rangle +\cdots +e^{\frac{2\pi i}{d}j\left(d-1\right)}|d-1\rangle ), \end{array}$$

where j=0,1,⋯,d−1. Similar to Ref. [89], the quantum Fourier transformation

(4) $$\begin{array}{c} H_{d}=\frac{1}{\sqrt{d}}\left(\begin{array}{cccc} 1 & 1 & \cdots & 1\\ 1 & e^{\frac{2\pi i}{d}1\cdot 1} & \cdots & e^{\frac{2\pi i}{d}1\cdot \left(d-1\right)}\\ \cdots & & & \cdots \\ 1 & e^{\frac{2\pi i}{d}\left(d-1\right)\cdot 1} & \cdots & e^{\frac{2\pi i}{d}\left(d-1\right)\cdot \left(d-1\right)} \end{array}\right) \end{array}$$

and the inverse quantum Fourier transformation

(5) $$\begin{array}{c} H_{d}^{-1}=\frac{1}{\sqrt{d}}\left(\begin{array}{cccc} 1 & 1 & \cdots & 1\\ 1 & e^{-\frac{2\pi i}{d}1\cdot 1} & \cdots & e^{-\frac{2\pi i}{d}\left(d-1\right)\cdot 1}\\ \cdots & & & \cdots \\ 1 & e^{-\frac{2\pi i}{d}1\cdot \left(d-1\right)} & \cdots & e^{-\frac{2\pi i}{d}\left(d-1\right)\cdot \left(d-1\right)} \end{array}\right) \end{array}$$

can implement transformation between eigenvectors |j〉 and ${|j\rangle }_{x}$(j=0,1,⋯,d−1):

(6) $$\begin{array}{c} {|j\rangle }_{x}=H_{d}|j\rangle ,|j\rangle =H_{d}^{-1}{|j\rangle }_{x}. \end{array}$$

The two-qudit C-NOT operation can be described as [68,89]:

(7) $$\begin{array}{c} U_{C}=\sum_{j_{1},j_{2}=0}^{d-1}|j_{1},j_{1}{⊕}_{d}j_{2}\rangle \langle j_{1},j_{2}|. \end{array}$$

Here $j_{1}{⊕}_{d}j_{2}$ means $j_{1}+j_{2}$ mod d.

Similar to the case of partially unknown operations of N qubits, the partially unknown operations of one qudit that have only one nonzero element in every row or every column of their representation matrices can be remotely implemented with fewer resources [49,50]. Since the unique nonzero element in the first row has d possible positions, the nonzero element in the second row has d−1 possible positions, and the nonzero element in the dth row has one possible position, there are d! restricted sets for the partially unknown operations of one qudit. The partially unknown operations of one qudit belonging to d! restricted sets, as suggested by Wang, can be described as [49,50]:

(8) $$\begin{array}{ccc} U_{l_{1},l_{2},\cdots ,l_{d-1}} & = & e^{i\varphi_{0{⊕}_{2}l_{1}{⊕}_{3}l_{2}{⊕}_{4}\cdots {⊕}_{d}l_{d-1}}}|0{⊕}_{2}l_{1}{⊕}_{3}l_{2}{⊕}_{4}\cdots {⊕}_{d}l_{d-1}\rangle \langle 0|\\ & + & e^{i\varphi_{1{⊕}_{2}l_{1}{⊕}_{3}l_{2}{⊕}_{4}\cdots {⊕}_{d}l_{d-1}}}|1{⊕}_{2}l_{1}{⊕}_{3}l_{2}{⊕}_{4}\cdots {⊕}_{d}l_{d-1}\rangle \langle 1|\\ & + & e^{i\varphi_{2{⊕}_{3}l_{2}{⊕}_{4}\cdots {⊕}_{d}l_{d-1}}}|2{⊕}_{3}l_{2}{⊕}_{4}\cdots {⊕}_{d}l_{d-1}\rangle \langle 2|\\ & + & \cdots +e^{i\varphi_{\left(d-1\right){⊕}_{d}l_{d-1}}}|\left(d-1\right){⊕}_{d}l_{d-1}\rangle \langle d-1|, \end{array}$$

where $l_{j}=0,1,\cdots ,j$(j=1,2,⋯,d−1) are used to label the d! restricted sets. $\varphi_{0},\varphi_{1},\cdots$, $\varphi_{d-1}$ are d real parameters. $\left(k-1\right){⊕}_{k}l_{k-1}$(k=2,⋯,d) means $\left(k-1\right)+l_{k-1}$ mod k. The n senders $Alice_{1},\cdots ,Alice_{n}$ share the information of the partially unknown operation $U_{l_{1},l_{2},\cdots ,l_{d-1}}\left(\varphi_{0},\varphi_{1},\cdots ,\varphi_{d-1}\right)$ to be remote implemented. That is, $Alice_{u}$(u=1,⋯,n) knows $\varphi_{u,0},\varphi_{u,1},\cdots ,\varphi_{u,d-1}$. Here,

(9) $$\begin{array}{ccc} \sum_{u=1}^{n}\varphi_{u,0} & = & \varphi_{0}\\ \sum_{u=1}^{n}\varphi_{u,1} & = & \varphi_{1}\\ & \cdots & \\ \sum_{u=1}^{n}\varphi_{u,d-1} & = & \varphi_{d-1}. \end{array}$$

all the senders $Alice_{1},\cdots ,Alice_{n}$ cooperate with each other to remotely implement the partially unknown operations and help the remote receiver to prepare the target state.

For the hierarchical joint remote implementation of partially unknown operations, the n senders $Alice_{1},\cdots ,$$Alice_{n}$, y upper-grade agents $Bob_{1},\cdots ,Bob_{y}$ and z lower-grade agents $Charlie_{1},\cdots ,Charlie_{z}$ share a (n + y + z)-qudit entangled state. One of the upper-grade agents $Bob_{1},\cdots ,Bob_{y}$ has a qudit b in an arbitrary state |ψ〉. The upper-grade agent first performs a C-NOT operation on his entangled particle and particle b, and then carries out a Z-basis measurement on particle b. The n senders $Alice_{1},\cdots ,$$Alice_{n}$ first perform corresponding unitary operations on their entangled particles according to the measurement result obtained by the upper-grade agent, and then implement partially unknown operations according to their information of the partially unknown operation to be remotely implemented. The upper-grade agents $Bob_{1},\cdots ,Bob_{y}$ can reconstruct the desired state with the cooperation of one of the lower-grade agents and the lower-grade agents $Charlie_{1},\cdots ,Charlie_{z}$ need the cooperation of all the other agents to prepare the desired state.

For the hierarchical joint remote implementation of partially unknown operations, all the agents share a (n + y + z)-qudit entangled state. The (n + y + z)-qudit entangled state shared by $Alice_{1},$⋯,$Alice_{n}$, $Bob_{1},\cdots ,Bob_{y}$$Charlie_{1},\cdots ,$$Charlie_{z}$ can be written as:

(10) $$\begin{array}{ccc} |\phi \rangle & = & \frac{1}{d}\sum_{j_{1},j_{2}=0}^{d-1}e^{\frac{2\pi i}{d}j_{1}j_{2}}{|j_{1},\cdots ,j_{1}\rangle }_{A_{1},\cdots ,A_{n}}\\ & & |j_{1},\cdots ,j_{1}\rangle_{B_{1},\cdots ,B_{y}}{|j_{2},\cdots ,j_{2}\rangle }_{C_{1},\cdots ,C_{z}}, \end{array}$$

where particles $A_{1},\cdots ,A_{n}$ belong to the sender $Alice_{1},$$\cdots ,Alice_{n}$, the upper-grade agents $Bob_{1},\cdots ,Bob_{y}$ are in possession of particles $B_{1},\cdots ,B_{y}$, and the lower-grade agents $Charlie_{1},\cdots ,Charlie_{z}$ are in possession of particles $C_{1},\cdots ,C_{z}$.

Without loss of generality, suppose $Bob_{1}$ has the qudit b in the arbitrary state [52]:

(11) $$\begin{array}{c} {|\psi \rangle }_{b}=\alpha_{0}|0\rangle +\alpha_{1}|1\rangle +\cdots +\alpha_{d-1}|d-1\rangle , \end{array}$$

where $|\alpha_{0}{|}^{2}+|\alpha_{1}{|}^{2}+\cdots +{\left|\alpha_{d-1}\right|}^{2}=1$. The n, senders $Alice_{1},\cdots ,Alice_{n}$ want to jointly remotely implement partially unknown operation $U_{l_{1},l_{2},\cdots ,l_{d-1}}$ and help the remote receiver prepare the target state $|\psi^{\prime }\rangle$.

(12) $$\begin{array}{ccc} |\psi^{\prime }\rangle & = & U_{l_{1},l_{2},\cdots ,l_{d-1}}|\psi \rangle \\ & = & e^{i\varphi_{0{⊕}_{2}l_{1}{⊕}_{3}l_{2}{⊕}_{4}\cdots {⊕}_{d}l_{d-1}}}\alpha_{0}|0{⊕}_{2}l_{1}{⊕}_{3}l_{2}{⊕}_{4}\cdots {⊕}_{d}l_{d-1}\rangle \\ & + & e^{i\varphi_{1{⊕}_{2}l_{1}{⊕}_{3}l_{2}{⊕}_{4}\cdots {⊕}_{d}l_{d-1}}}\alpha_{1}|1{⊕}_{2}l_{1}{⊕}_{3}l_{2}{⊕}_{4}\cdots {⊕}_{d}l_{d-1}\rangle \\ & + & e^{i\varphi_{2{⊕}_{3}l_{2}{⊕}_{4}\cdots {⊕}_{d}l_{d-1}}}\alpha_{2}|2{⊕}_{3}l_{2}{⊕}_{4}\cdots {⊕}_{d}l_{d-1}\rangle \\ & + & \cdots +e^{i\varphi_{\left(d-1\right){⊕}_{d}l_{d-1}}}\alpha_{d-1}|\left(d-1\right){⊕}_{d}l_{d-1}\rangle . \end{array}$$

The state of particles $A_{1},\cdots ,A_{n}$, $B_{1},\cdots ,B_{y}$, $C_{1},\cdots ,$$C_{z}$,b can be written as:

(13) $$\begin{array}{ccc} |\Phi \rangle & = & {|\phi \rangle }_{A_{1},\cdots ,A_{n},B_{1},\cdots ,B_{y},C_{1},\cdots ,C_{z}}⊗{|\psi \rangle }_{b}\\ & = & \frac{1}{d}\sum_{j_{1},j_{2},j_{3}=0}^{d-1}e^{\frac{2\pi i}{d}j_{1}j_{2}}\alpha_{j_{3}}|j_{1},\cdots ,j_{1}\rangle_{A_{1},\cdots ,A_{n}}{|j_{1},\cdots ,j_{1}\rangle }_{B_{1},\cdots ,B_{y}}\\ & & |j_{2},\cdots ,j_{2}\rangle_{C_{1},\cdots ,C_{z}}{|j_{3}\rangle }_{b}. \end{array}$$

For the hierarchical joint remote implementation of the partially unknown operation $U_{l_{1},l_{2},\cdots ,l_{d-1}}$, $Bob_{1}$ first implements C-NOT operation on qudits $B_{1}$ and b by using qudit $B_{1}$ as the control qudit, and then performs Z-basis measurement on qudit b. After the C-NOT operation, the state of particles $A_{1},\cdots ,A_{n}$, $B_{1},\cdots ,B_{y}$, $C_{1},\cdots ,$$C_{z}$, and b becomes:

(14) $$\begin{array}{ccc} |\Phi_{1}\rangle & = & \frac{1}{d}\sum_{j_{1},j_{2},j_{3}=0}^{d-1}e^{\frac{2\pi i}{d}j_{1}j_{2}}\alpha_{j_{3}}|j_{1},\cdots ,j_{1}\rangle_{A_{1},\cdots ,A_{n}}{|j_{1},\cdots ,j_{1}\rangle }_{B_{1},\cdots ,B_{y}}\\ & & |j_{2},\cdots ,j_{2}\rangle_{C_{1},\cdots ,C_{z}}{|j_{1}{⊕}_{d}j_{3}\rangle }_{b}. \end{array}$$

The state of particles $A_{1},\cdots ,A_{n}$, $B_{1},\cdots ,B_{y}$, $C_{1},\cdots ,$$C_{z}$ becomes ${|\phi \rangle }_{1}$ if the Z-basis measurement result is t (t=0,1,⋯,d−1).

(15) $$\begin{array}{ccc} |\phi_{1}\rangle & = & \frac{1}{\sqrt{d}}\sum_{j_{1},j_{2}=0}^{d-1}e^{\frac{2\pi i}{d}j_{1}j_{2}}\alpha_{t{⊕}_{d}\left(d-j_{1}\right)}{|j_{1},\cdots ,j_{1}\rangle }_{A_{1},\cdots ,A_{n}}\\ & & |j_{1},\cdots ,j_{1}\rangle_{B_{1},\cdots ,B_{y}}{|j_{2},\cdots ,j_{2}\rangle }_{C_{1},\cdots ,C_{z}}. \end{array}$$

To implement partially unknown operations remotely, $Alice_{u}$(u=1,⋯,n) first implements single qudit operation $X_{t}$ on its qudit $A_{u}$ according to the Z-basis measurement result t, and then implements partially unknown operation $U_{l_{1},l_{2},\cdots ,l_{d-1}}\left(\varphi_{u,0},\varphi_{u,1},\cdots ,\varphi_{u,d-1}\right)$ in accordance with its information of $\varphi_{u,0},\varphi_{u,1},\cdots ,\varphi_{u,d-1}$. The single qudit operation $X_{t}$ in accordance with the Z-basis measurement result t can be written as:

(16) $$\begin{array}{c} X_{t}=\sum_{j=0}^{d-1}|t{⊕}_{d}\left(d-j\right)\rangle \langle j|. \end{array}$$

The state of particles $A_{1},\cdots ,A_{n}$, $B_{1},\cdots ,B_{y}$, $C_{1},\cdots ,$$C_{z}$ becomes ${|\phi \rangle }_{2}$ after $Alice_{u}$(u=1,⋯,n) implements single qudit operation $X_{t}$ on its qudit $A_{u}$.

(17) $$\begin{array}{ccc} |\phi_{2}\rangle & = & \frac{1}{\sqrt{d}}\sum_{j_{1},j_{2}=0}^{d-1}e^{\frac{2\pi i}{d}\left(t-j_{1}\right)j_{2}}\alpha_{j_{1}}{|j_{1},\cdots ,j_{1}\rangle }_{A_{1},\cdots ,A_{n}}\\ & & |t{⊕}_{d}\left(d-j_{1}\right),\cdots ,t{⊕}_{d}\left(d-j_{1}\right)\rangle_{B_{1},\cdots ,B_{y}}\\ & & |j_{2},\cdots ,j_{2}\rangle_{C_{1},\cdots ,C_{z}}. \end{array}$$

After the single qudit operation $X_{t}$, $Alice_{u}$(u=1,⋯,n) implements partially unknown operation $U_{l_{1},l_{2},\cdots ,l_{d-1}}\left(\varphi_{u,0},\varphi_{u,1},\cdots ,\varphi_{u,d-1}\right)$ in accordance with its information of $\varphi_{u,0},\varphi_{u,1},\cdots ,\varphi_{u,d-1}$ on qudit $A_{u}$. The state of particles $A_{1},\cdots ,A_{n}$, $B_{1},\cdots ,B_{y}$, $C_{1},\cdots ,$$C_{z}$ becomes (without normalization):

(18) $$\begin{array}{ccc} |\phi_{3}\rangle & = & \sum_{j_{2}=0}^{d-1}|j_{2},\cdots ,j_{2}\rangle_{C_{1},\cdots ,C_{z}}[\alpha_{0}e^{\frac{2\pi i}{d}tj_{2}}e^{i(\varphi_{1,0{⊕}_{2}l_{1}\cdots {⊕}_{d}l_{d-1}}+\cdots +\varphi_{n,0{⊕}_{2}l_{1}\cdots {⊕}_{d}l_{d-1}})}\\ & & |0{⊕}_{2}l_{1}\cdots {⊕}_{d}l_{d-1},\cdots ,0{⊕}_{2}l_{1}\cdots {⊕}_{d}l_{d-1}\rangle |t,\cdots ,t\rangle \\ & + & \alpha_{1}e^{\frac{2\pi i}{d}\left(t+d-1\right)j_{2}}e^{i(\varphi_{1,1{⊕}_{2}l_{1}\cdots {⊕}_{d}l_{d-1}}+\cdots +\varphi_{n,1{⊕}_{2}l_{1}\cdots {⊕}_{d}l_{d-1}})}\\ & & |1{⊕}_{2}l_{1}\cdots {⊕}_{d}l_{d-1},\cdots ,1{⊕}_{2}l_{1}\cdots {⊕}_{d}l_{d-1}\rangle \\ & & |t{⊕}_{d}\left(d-1\right),\cdots ,t{⊕}_{d}\left(d-1\right)\rangle +\cdots \\ & + & \alpha_{d-1}e^{\frac{2\pi i}{d}\left(t+1\right)j_{2}}e^{i(\varphi_{1,\left(d-1\right){⊕}_{d}l_{d-1}}+\cdots +\varphi_{n,\left(d-1\right){⊕}_{d}l_{d-1}})}\\ & & |\left(d-1\right){⊕}_{d}l_{d-1},\cdots ,\left(d-1\right){⊕}_{d}l_{d-1}\rangle \\ & & |t{⊕}_{d}1,\cdots ,t{⊕}_{d}1{\rangle ]}_{A_{1},\cdots ,A_{n},B_{1},\cdots ,B_{y}}\\ & = & \sum_{j_{2}=0}^{d-1}|j_{2},\cdots ,j_{2}\rangle_{C_{1},\cdots ,C_{z}}[\alpha_{0}e^{\frac{2\pi i}{d}tj_{2}}e^{i\varphi_{0{⊕}_{2}l_{1}\cdots {⊕}_{d}l_{d-1}}}\\ & & |0{⊕}_{2}l_{1}\cdots {⊕}_{d}l_{d-1},\cdots ,0{⊕}_{2}l_{1}\cdots {⊕}_{d}l_{d-1}\rangle |t,\cdots ,t\rangle \\ & + & \alpha_{1}e^{\frac{2\pi i}{d}\left(t+d-1\right)j_{2}}e^{i\varphi_{1{⊕}_{2}l_{1}\cdots {⊕}_{d}l_{d-1}}}\\ & & |1{⊕}_{2}l_{1}\cdots {⊕}_{d}l_{d-1},\cdots ,1{⊕}_{2}l_{1}\cdots {⊕}_{d}l_{d-1}\rangle \\ & & |t{⊕}_{d}\left(d-1\right),\cdots ,t{⊕}_{d}\left(d-1\right)\rangle +\cdots \\ & + & \alpha_{d-1}e^{\frac{2\pi i}{d}\left(t+1\right)j_{2}}e^{i\varphi_{\left(d-1\right){⊕}_{d}l_{d-1}}}|\left(d-1\right){⊕}_{d}l_{d-1},\cdots ,\left(d-1\right){⊕}_{d}l_{d-1}\rangle \\ & & |t{⊕}_{d}1,\cdots ,t{⊕}_{d}1{\rangle ]}_{A_{1},\cdots ,A_{n},B_{1},\cdots ,B_{y}}. \end{array}$$

To jointly remotely implement the partially unknown operation, $Alice_{u}$(u=1,⋯,n) performs X-basis measurement on his entangled particle $A_{u}$. The state of particles $A_{1},\cdots$, $A_{n},B_{1},\cdots ,B_{y},C_{1},\cdots ,C_{z}$ can be rewritten as (neglecting the whole factor):

(19) $$\begin{array}{ccc} |\phi_{3}\rangle & = & \sum_{r_{1},\cdots ,r_{n},j_{2}=0}^{d-1}|j_{2},\cdots ,j_{2}\rangle_{C_{1},\cdots ,C_{z}}[\alpha_{0}e^{\frac{2\pi i}{d}tj_{2}}\\ & & e^{i\varphi_{0{⊕}_{2}l_{1}\cdots {⊕}_{d}l_{d-1}}}e^{-\frac{2\pi i}{d}\left(r_{1}+\cdots +r_{n}\right)\left(0{⊕}_{2}l_{1}\cdots {⊕}_{d}l_{d-1}\right)}\\ & & |r_{1}\rangle_{x}⊗\cdots ⊗|r_{n}\rangle_{x}|t,\cdots ,t\rangle +\alpha_{1}e^{\frac{2\pi i}{d}\left(t+d-1\right)j_{2}}\\ & & e^{i\varphi_{1{⊕}_{2}l_{1}\cdots {⊕}_{d}l_{d-1}}}e^{-\frac{2\pi i}{d}\left(r_{1}+\cdots +r_{n}\right)\left(1{⊕}_{2}l_{1}\cdots {⊕}_{d}l_{d-1}\right)}\\ & & |r_{1}\rangle_{x}⊗\cdots ⊗|r_{n}\rangle_{x}|t{⊕}_{d}\left(d-1\right),\cdots ,t{⊕}_{d}\left(d-1\right)\rangle \\ & & +\cdots +\alpha_{d-1}e^{\frac{2\pi i}{d}\left(t+1\right)j_{2}}e^{i\varphi_{\left(d-1\right){⊕}_{d}l_{d-1}}}\\ & & e^{-\frac{2\pi i}{d}\left(r_{1}+\cdots +r_{n}\right)\left(\left(d-1\right){⊕}_{d}l_{d-1}\right)}|r_{1}\rangle_{x}⊗\cdots ⊗{|r_{n}\rangle }_{x}\\ & & |t{⊕}_{d}1,\cdots ,t{⊕}_{d}1{\rangle ]}_{A_{1},\cdots ,A_{n},B_{1},\cdots ,B_{y}}. \end{array}$$

The state of particles $B_{1},\cdots ,B_{y},C_{1},\cdots ,C_{z}$ becomes $|\phi_{4}\rangle$ if the measurement result obtained by $A_{u}$(u=1,⋯,n) is $|r_{u}\rangle_{x}$$\left(r_{u}=0,\cdots ,d-1\right)$.

(20) $$\begin{array}{ccc} |\phi_{4}\rangle & = & \sum_{j_{2}=0}^{d-1}|j_{2},\cdots ,j_{2}\rangle_{C_{1},\cdots ,C_{z}}[\alpha_{0}e^{\frac{2\pi i}{d}tj_{2}}\\ & & e^{i\varphi_{0{⊕}_{2}l_{1}\cdots {⊕}_{d}l_{d-1}}}e^{-\frac{2\pi i}{d}\left(0{⊕}_{2}l_{1}\cdots {⊕}_{d}l_{d-1}\right)r}|t,\cdots ,t\rangle \\ & + & \alpha_{1}e^{\frac{2\pi i}{d}\left(t+d-1\right)j_{2}}e^{i\varphi_{1{⊕}_{2}l_{1}\cdots {⊕}_{d}l_{d-1}}}e^{-\frac{2\pi i}{d}\left(1{⊕}_{2}l_{1}\cdots {⊕}_{d}l_{d-1}\right)r}\\ & & |t{⊕}_{d}\left(d-1\right),\cdots ,t{⊕}_{d}\left(d-1\right)\rangle +\cdots \\ & + & \alpha_{d-1}e^{\frac{2\pi i}{d}\left(t+1\right)j_{2}}e^{i\varphi_{\left(d-1\right){⊕}_{d}l_{d-1}}}e^{-\frac{2\pi i}{d}\left(\left(d-1\right){⊕}_{d}l_{d-1}\right)r}\\ & & |t{⊕}_{d}1,\cdots ,t{⊕}_{d}1{\rangle ]}_{B_{1},\cdots ,B_{y}}, \end{array}$$

where $r=r_{1}+\cdots +r_{n}$.

**Figure 1:**
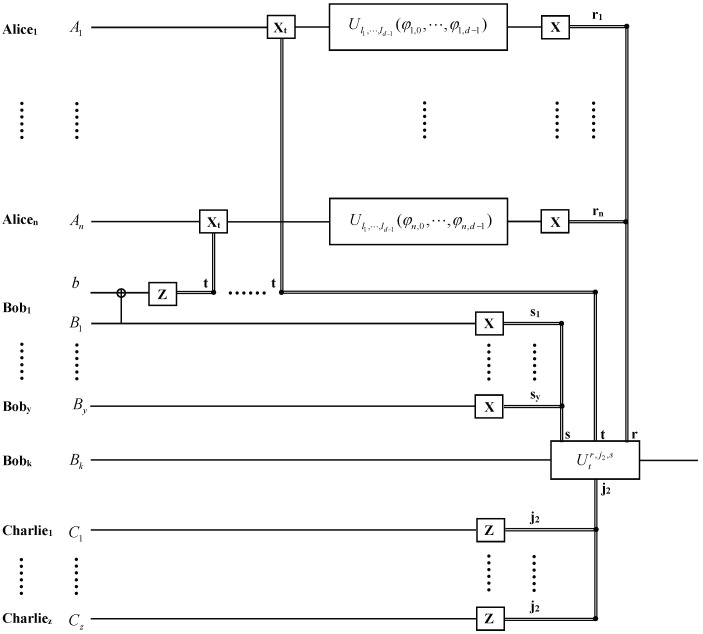
The quantum circuit for the hierarchial joint remote implementation of partially unknown operations of one qudit with upper-grade agent $Bob_{k}$. Classical communication from the sender $Alice_{j}$(j=1,⋯,n), the upper-grade agents $Bob_{1},\cdots ,Bob_{k-1},Bob_{k+1},\cdots ,Bob_{y}$ and the the lower-grade agent $Charlie_{p}$(p=1,⋯,z) to the receiver $Bob_{k}$ are represented by double lines. $r_{1},\cdots ,r_{n}$, $s_{1},\cdots ,s_{y}$ and t, $j_{2}$ denote the results of the generalized X-basis measurements and generalized Z-basis measurements. $X_{t}$, $U_{l_{1},\cdots ,l_{d-1}}\left(\varphi_{j,0},\cdots ,\varphi_{j,d-1}\right)$ in the solid-line boxes denote the single-qudit operations performed by the sender $Alice_{j}$, and $U_{t}^{r,j_{2},s}$ in the solid-line box denotes the unitary operation performed by the receiver.

Firstly, we suppose that the agents agree to reconstruct the desired state $|\psi^{\prime }\rangle$ at the upper-grade agent $Bob_{k}$’s site (k=1,⋯,y). The quantum circuit for the hierarchial joint remote implementation of partially unknown operations of one qudit with upper-grade agent is shown in Figure 1. To jointly remotely implement the partially unknown operations, the lower-grade agent $Charlie_{p}$(p=1,⋯,z) performs a Z-basis measurement on its qudit $C_{p}$. The upper-grade agents $Bob_{1},\cdots ,Bob_{k-1},Bob_{k+1},\cdots ,Bob_{y}$ perform X-basis measurements on qudits $B_{1},\cdots ,B_{k-1},B_{k+1},\cdots ,B_{y}$. The receiver $Bob_{k}$ can prepare the target state $|\psi^{\prime }\rangle$ by cooperating with one of the lower-grade agents.

After the Z-basis measurement, the state of particles $B_{1},\cdots ,B_{y}$ becomes $|\phi_{5}\rangle$ if the measurement result obtained by $Charlie_{p}$(p=1,⋯,z) is $|j_{2}\rangle$$\left(j_{2}=0,\cdots ,d-1\right)$.

(21) $$\begin{array}{ccc} |\phi_{5}\rangle & = & [\alpha_{0}e^{\frac{2\pi i}{d}tj_{2}}e^{i\varphi_{0{⊕}_{2}l_{1}\cdots {⊕}_{d}l_{d-1}}}e^{-\frac{2\pi i}{d}\left(0{⊕}_{2}l_{1}\cdots {⊕}_{d}l_{d-1}\right)r}|t,\cdots ,t\rangle \\ & + & \alpha_{1}e^{\frac{2\pi i}{d}\left(t+d-1\right)j_{2}}e^{i\varphi_{1{⊕}_{2}l_{1}\cdots {⊕}_{d}l_{d-1}}}e^{-\frac{2\pi i}{d}\left(1{⊕}_{2}l_{1}\cdots {⊕}_{d}l_{d-1}\right)r}\\ & & |t{⊕}_{d}\left(d-1\right),\cdots ,t{⊕}_{d}\left(d-1\right)\rangle +\cdots \\ & + & \alpha_{d-1}e^{\frac{2\pi i}{d}\left(t+1\right)j_{2}}e^{i\varphi_{\left(d-1\right){⊕}_{d}l_{d-1}}}e^{-\frac{2\pi i}{d}\left(\left(d-1\right){⊕}_{d}l_{d-1}\right)r}\\ & & |t{⊕}_{d}1,\cdots ,t{⊕}_{d}1{\rangle ]}_{B_{1},\cdots ,B_{y}}. \end{array}$$

To implement the partially unknown operations remotely, the upper-grade agents $Bob_{1},\cdots ,Bob_{k-1},$$Bob_{k+1},\cdots ,Bob_{y}$ perform X-basis measurements on their qudits $B_{1},\cdots$, $B_{k-1},B_{k+1},\cdots ,B_{y}$. The state of particles $B_{1},\cdots ,B_{y}$ can be rewritten as:

(22) |ϕ6〉=∑s1,⋯,sk−1,sk+1,⋯,sy=0d−1[α0e−2πid(s1+⋯+sk−1+sk+1+⋯+sy)te2πidtj2eiφ0⊕2l1⋯⊕dld−1e−2πid(0⊕2l1⋯⊕dld−1)r|s1〉x⊗⋯⊗|sk−1〉x|t〉|sk+1〉x⊗⋯⊗|sy〉x+α1e−2πid(s1+⋯+sk−1+sk+1+⋯+sy)(t+d−1)e2πid(t+d−1)j2eiφ1⊕2l1⋯⊕dld−1e−2πid(1⊕2l1⋯⊕dld−1)r|s1〉x⊗⋯⊗|sk−1〉x|t⊕d(d−1)〉|sk+1〉x⊗⋯⊗|sy〉x+⋯+αd−1e−2πid(s1+⋯+sk−1+sk+1+⋯+sy)(t+1)e2πid(t+1)j2eiφ(d−1)⊕dld−1e−2πid((d−1)⊕dld−1)r|s1〉x⊗⋯⊗|sk−1〉x|t⊕d1〉|sk+1〉x⊗⋯⊗|sy〉x]B1,⋯,Bk−1,Bk,Bk+1,⋯,By.

The state of particle $B_{k}$ becomes ${|\Psi \rangle }_{B_{k}}$ if the measurement results obtained by $Bob_{1},\cdots$, $Bob_{k-1},Bob_{k+1},$$\cdots ,Bob_{y}$ are $|s_{1}\rangle_{x},\cdots ,|s_{k-1}\rangle_{x},{|s_{k+1}\rangle }_{x},\cdots ,$$|s_{y}\rangle_{x}$.

(23) $$\begin{array}{ccc} {|\Psi \rangle }_{B_{k}} & = & [\alpha_{0}e^{-\frac{2\pi i}{d}st}e^{\frac{2\pi i}{d}tj_{2}}e^{i\varphi_{0{⊕}_{2}l_{1}\cdots {⊕}_{d}l_{d-1}}}e^{-\frac{2\pi i}{d}\left(0{⊕}_{2}l_{1}\cdots {⊕}_{d}l_{d-1}\right)r}|t\rangle \\ & + & \alpha_{1}e^{-\frac{2\pi i}{d}s\left(t+d-1\right)}e^{\frac{2\pi i}{d}\left(t+d-1\right)j_{2}}e^{i\varphi_{1{⊕}_{2}l_{1}\cdots {⊕}_{d}l_{d-1}}}\\ & & e^{-\frac{2\pi i}{d}\left(1{⊕}_{2}l_{1}\cdots {⊕}_{d}l_{d-1}\right)r}|t{⊕}_{d}\left(d-1\right)\rangle +\cdots \\ & + & \alpha_{d-1}e^{-\frac{2\pi i}{d}s\left(t+1\right)}e^{\frac{2\pi i}{d}\left(t+1\right)j_{2}}e^{i\varphi_{\left(d-1\right){⊕}_{d}l_{d-1}}}\\ & & e^{-\frac{2\pi i}{d}\left(\left(d-1\right){⊕}_{d}l_{d-1}\right)r}|t{⊕}_{d}1{\rangle ]}_{B_{k}}, \end{array}$$

where $s=s_{1}+\cdots +s_{k-1}+s_{k+1}+\cdots +s_{y}$.

The single-qudit operation

(24) $$\begin{array}{ccc} U_{t}^{r,j_{2},s} & = & e^{\frac{2\pi i}{d}\left(s-j_{2}\right)t}e^{\frac{2\pi i}{d}\left(0{⊕}_{2}l_{1}\cdots {⊕}_{d}l_{d-1}\right)r}|0{⊕}_{2}l_{1}\cdots {⊕}_{d}l_{d-1}\rangle \langle t|\\ & + & e^{\frac{2\pi i}{d}\left(s-j_{2}\right)\left(t+d-1\right)}e^{\frac{2\pi i}{d}\left(1{⊕}_{2}l_{1}\cdots {⊕}_{d}l_{d-1}\right)r}\\ & & |1{⊕}_{2}l_{1}\cdots {⊕}_{d}l_{d-1}\rangle \langle t{⊕}_{d}\left(d-1\right)|+\cdots \\ & + & e^{\frac{2\pi i}{d}\left(s-j_{2}\right)\left(t+1\right)}e^{\frac{2\pi i}{d}\left(\left(d-1\right){⊕}_{d}l_{d-1}\right)r}\\ & & |\left(d-1\right){⊕}_{d}l_{d-1}\rangle \langle t{⊕}_{d}1| \end{array}$$

in accordance with measurement results $t,r,j_{2},s$ can transform state ${|\Psi \rangle }_{B_{k}}$ to the target state $|\psi^{\prime }\rangle$:

(25) $$\begin{array}{c} |\psi^{\prime }\rangle_{B_{k}}=U_{t}^{r,j_{2},s}{|\Psi \rangle }_{B_{k}}. \end{array}$$

Similar to the hierarchical remote implementation of partially unknown operation of one qubit [61], the upper-grade agent needs to only cooperate with one of the lower-grade agents to prepare the target state.

**Figure 2:**
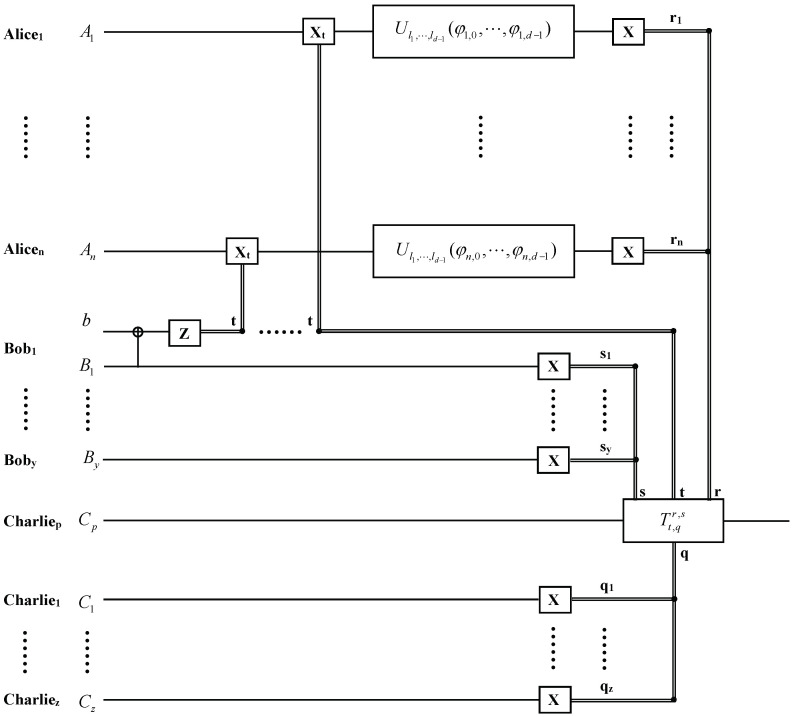
The quantum circuit for the hierarchial joint remote implementation of partially unknown operations of one qudit with lower-grade agent $Charlie_{p}$. $r_{1},\cdots ,r_{n}$, $s_{1},\cdots ,s_{y}$, $q_{1},\cdots ,q_{z}$ and t, $j_{2}$ are the results of the generalized X-basis measurements and generalized Z-basis measurements. $T_{t,q}^{r,s}$ in the solid-line box denotes the unitary operation performed by the receiver $Charlie_{p}$.

Now, we discuss another case, in which all of the agents agree to reconstruct the desired state $|\psi^{\prime }\rangle$ at the lower-grade agent $Charlie_{p}$’s site (p=1,⋯,z). The quantum circuit for the hierarchial joint remote implementation of partially unknown operations of one qudit with lower-grade agent is shown in Figure 2. To reconstruct the desired state $|\psi^{\prime }\rangle$ at the lower-grade agent $Charlie_{p}$’s site, the other lower-grade agents $Charlie_{1},\cdots$, $Charlie_{p-1},Charlie_{p+1},\cdots ,Charlie_{z}$ and the upper-grade agents $Bob_{1},\cdots ,Bob_{y}$ perform X-basis measurements on their entangled particles $C_{1},\cdots ,C_{p-1},C_{p+1},\cdots ,C_{z}$ and $B_{1},\cdots ,B_{y}$, after $Alice_{u}$(u=1,⋯,n) implements the partially unknown operation $U_{l_{1},l_{2},\cdots ,l_{d-1}}(\varphi_{l,0},\varphi_{l,1},\cdots$, $\varphi_{l,d-1})$ and performs an x-basis measurement on qudit $A_{u}$. The state $|\phi_{4}\rangle$ can be rewritten as:

(26) |ϕ4〉C1,⋯,Cp−1,Cp+1,⋯,Cz,B1,⋯,By,Cp=∑q1,⋯,qp−1,qp+1,⋯,qz,s1,⋯,sy=0d−1|q1〉x⊗⋯⊗|qp−1〉x⊗|qp+1〉x⊗⋯⊗|qz〉x|s1〉x⊗⋯⊗|sy〉x{α0eiφ0⊕2l1⋯⊕dld−1e−2πid(0⊕2l1⋯⊕dld−1)re−2πidts[∑j2=0d−1e2πid(t−q)j2|j2〉]+α1eiφ1⊕2l1⋯⊕dld−1e−2πid(1⊕2l1⋯⊕dld−1)re−2πid(t+d−1)s[∑j2=0d−1e2πid(t+d−1−q)j2|j2〉]+⋯+αd−1eiφ(d−1)⊕dld−1e−2πid((d−1)⊕dld−1)re−2πid(t+1)s[∑j2=0d−1e2πid(t+1−q)j2|j2〉]}.

Here, $q=q_{1}+\cdots +q_{p-1}+q_{p+1}+\cdots +q_{z}$.

The state of particle $C_{p}$ becomes ${|\omega \rangle }_{C_{p}}$ if the measurement results obtained by $Charlie_{1}$, $\cdots ,Charlie_{p-1},$$Charlie_{p+1},\cdots ,Charlie_{z}$ and $Bob_{1},\cdots ,Bob_{y}$ are $|q_{1}\rangle_{x},$$\cdots ,|q_{p-1}\rangle_{x},{|q_{p+1}\rangle }_{x}$, ⋯,$|q_{z}\rangle_{x}$ and $|s_{1}\rangle_{x},\cdots ,{|s_{y}\rangle }_{x}$.

(27) $$\begin{array}{ccc} {|\omega \rangle }_{C_{p}} & = & \alpha_{0}e^{i\varphi_{0{⊕}_{2}l_{1}\cdots {⊕}_{d}l_{d-1}}}e^{-\frac{2\pi i}{d}\left(0{⊕}_{2}l_{1}\cdots {⊕}_{d}l_{d-1}\right)r}\\ & & e^{-\frac{2\pi i}{d}ts}[\sum_{j_{2}=0}^{d-1}e^{\frac{2\pi i}{d}\left(t-q\right)j_{2}}|j_{2}\rangle ]\\ & + & \alpha_{1}e^{i\varphi_{1{⊕}_{2}l_{1}\cdots {⊕}_{d}l_{d-1}}}e^{-\frac{2\pi i}{d}\left(1{⊕}_{2}l_{1}\cdots {⊕}_{d}l_{d-1}\right)r}\\ & & e^{-\frac{2\pi i}{d}\left(t+d-1\right)s}[\sum_{j_{2}=0}^{d-1}e^{\frac{2\pi i}{d}\left(t+d-1-q\right)j_{2}}|j_{2}\rangle ]+\cdots \\ & + & \alpha_{d-1}e^{i\varphi_{\left(d-1\right){⊕}_{d}l_{d-1}}}e^{-\frac{2\pi i}{d}\left(\left(d-1\right){⊕}_{d}l_{d-1}\right)r}\\ & & e^{-\frac{2\pi i}{d}\left(t+1\right)s}[\sum_{j_{2}=0}^{d-1}e^{\frac{2\pi i}{d}\left(t+1-q\right)j_{2}}|j_{2}\rangle ]. \end{array}$$

To reconstruct the desired state $|\psi^{\prime }\rangle$, $Charlie_{p}$ first applies the inverse quantum Fourier transformation on its particle $C_{p}$, and then applies corresponding unitary operation on its particle in accordance with all the other agents measurement results. The inverse quantum Fourier transformation $H_{d}^{-1}$ transform quantum state ${|\omega \rangle }_{C_{p}}$ to

(28) $$\begin{array}{ccc} |\omega_{1}\rangle_{C_{p}} & = & \alpha_{0}e^{i\varphi_{0{⊕}_{2}l_{1}\cdots {⊕}_{d}l_{d-1}}}e^{-\frac{2\pi i}{d}\left(0{⊕}_{2}l_{1}\cdots {⊕}_{d}l_{d-1}\right)r}\\ & & e^{-\frac{2\pi i}{d}ts}H_{d}^{-1}[\sum_{j_{2}=0}^{d-1}e^{\frac{2\pi i}{d}\left(t-q\right)j_{2}}|j_{2}\rangle ]\\ & + & \alpha_{1}e^{i\varphi_{1{⊕}_{2}l_{1}\cdots {⊕}_{d}l_{d-1}}}e^{-\frac{2\pi i}{d}\left(1{⊕}_{2}l_{1}\cdots {⊕}_{d}l_{d-1}\right)r}\\ & & e^{-\frac{2\pi i}{d}\left(t+d-1\right)s}H_{d}^{-1}[\sum_{j_{2}=0}^{d-1}e^{\frac{2\pi i}{d}\left(t+d-1-q\right)j_{2}}|j_{2}\rangle ]+\cdots \\ & + & \alpha_{d-1}e^{i\varphi_{\left(d-1\right){⊕}_{d}l_{d-1}}}e^{-\frac{2\pi i}{d}\left(\left(d-1\right){⊕}_{d}l_{d-1}\right)r}\\ & & e^{-\frac{2\pi i}{d}\left(t+1\right)s}H_{d}^{-1}[\sum_{j_{2}=0}^{d-1}e^{\frac{2\pi i}{d}\left(t+1-q\right)j_{2}}|j_{2}\rangle ]\\ & = & \alpha_{0}e^{i\varphi_{0{⊕}_{2}l_{1}\cdots {⊕}_{d}l_{d-1}}}e^{-\frac{2\pi i}{d}\left(0{⊕}_{2}l_{1}\cdots {⊕}_{d}l_{d-1}\right)r}\\ & & e^{-\frac{2\pi i}{d}ts}|t{⊕}_{d}\left(d-q\right)\rangle \\ & + & \alpha_{1}e^{i\varphi_{1{⊕}_{2}l_{1}\cdots {⊕}_{d}l_{d-1}}}e^{-\frac{2\pi i}{d}\left(1{⊕}_{2}l_{1}\cdots {⊕}_{d}l_{d-1}\right)r}\\ & & e^{-\frac{2\pi i}{d}\left(t+d-1\right)s}|t{⊕}_{d}\left(d-1-q\right)\rangle +\cdots \\ & + & \alpha_{d-1}e^{i\varphi_{\left(d-1\right){⊕}_{d}l_{d-1}}}e^{-\frac{2\pi i}{d}\left(\left(d-1\right){⊕}_{d}l_{d-1}\right)r}\\ & & e^{-\frac{2\pi i}{d}\left(t+1\right)s}|t{⊕}_{d}\left(1-q\right)\rangle . \end{array}$$

The unitary operation $T_{t,q}^{r,s}$

(29) $$\begin{array}{ccc} T_{t,q}^{r,s} & = & e^{\frac{2\pi i}{d}\left(0{⊕}_{2}l_{1}\cdots {⊕}_{d}l_{d-1}\right)r}e^{\frac{2\pi i}{d}ts}\\ & & |0{⊕}_{2}l_{1}\cdots {⊕}_{d}l_{d-1}\rangle \langle t{⊕}_{d}\left(d-q\right)|\\ & + & e^{\frac{2\pi i}{d}\left(1{⊕}_{2}l_{1}\cdots {⊕}_{d}l_{d-1}\right)r}e^{\frac{2\pi i}{d}\left(t+d-1\right)s}\\ & & |1{⊕}_{2}l_{1}\cdots {⊕}_{d}l_{d-1}\rangle \langle t{⊕}_{d}\left(d-1-q\right)|+\cdots \\ & + & e^{\frac{2\pi i}{d}\left(\left(d-1\right){⊕}_{d}l_{d-1}\right)r}e^{\frac{2\pi i}{d}\left(t+1\right)s}\\ & & |\left(d-1\right){⊕}_{d}l_{d-1}\rangle \langle t{⊕}_{d}\left(1-q\right)| \end{array}$$

in accordance with the senders’ measurement results r and t, the upper-grade agents’ measurement results and the other lower-grade agents’ measurement result q will transform state $|\omega_{1}\rangle_{C_{p}}$ to the target state ${|\psi \rangle }_{C_{p}}$.

(30) $$\begin{array}{c} {|\psi \rangle }_{C_{p}}=T_{t,q}^{r,s}{|\omega_{1}\rangle }_{C_{p}}. \end{array}$$

The partially unknown operations of one qudit can be remotely implemented via local unitary operations, classical communication, and one multiparticle entangled state. Compared to previously protocol for remotely implementing partially unknown operations of one qubit, the protocol has the advantage of having a high channel capacity by transmitting d coefficients $\varphi_{0},\cdots ,\varphi_{d-1}$ via one multiparticle entangled state [48].

## 3. Hierarchial Joint Remote Implementation of Partially Unknown Operations of m Qudit via m Multiparticle High-Dimensional Entangled States

Now, let us generalize the protocol for the hierarchial joint remote implementation of partially unknown operations of m qudit with m multiparticle high-dimensional entangled states. The senders share the information of the partially unknown operations to be remotely implemented and cooperate with each other to help the remote receiver to reconstruct the desired state.

Similar to the case for the remote implementation of partially unknown operations of one qudit, the partially unknown operations of m qudits have only one nonzero element in every row or every column of their representation matrices. There are M!$\left(M=d^{m}\right)$ restricted sets for partially unknown operations of m qudits, since the unique nonzero element in the first row has M possible positions, the nonzero element in the second row has M−1 possible positions, and the nonzero element in the Mth row has one possible position. The partially unknown operations of m qudit belongs to M! restricted sets as suggested by Wang can be described as [50]:

(31) $$\begin{array}{ccc} U_{l_{1},l_{2},\cdots ,l_{M}} & = & e^{i\varphi_{0{⊕}_{2}l_{1}{⊕}_{3}l_{2}{⊕}_{4}\cdots {⊕}_{M}l_{M-1}}}|t_{1}^{0},\cdots ,t_{m}^{0}\rangle \langle 0,\cdots ,0|\\ & + & e^{i\varphi_{1{⊕}_{2}l_{1}{⊕}_{3}l_{2}{⊕}_{4}\cdots {⊕}_{M}l_{M-1}}}|t_{1}^{1},\cdots ,t_{m}^{1}\rangle \langle 0,\cdots ,1|\\ & + & e^{i\varphi_{2{⊕}_{3}l_{2}{⊕}_{4}\cdots {⊕}_{M}l_{M-1}}}|t_{1}^{2},\cdots ,t_{m}^{2}\rangle \langle 0,\cdots ,2|+\cdots \\ & + & e^{i\varphi_{\left(M-1\right){⊕}_{M}l_{M-1}}}|t_{1}^{M-1},\cdots ,t_{m}^{M-1}\rangle \langle d-1,\cdots ,d-1|, \end{array}$$

where $\varphi_{0},\varphi_{1},\cdots ,\varphi_{M-1}$ are M real parameters, $l_{j}=0,1,\cdots ,j$(j=1,2,⋯,M−1) are used to label the M! restricted sets. $t_{j}^{l}=0,\cdots ,d-1$(j=1,⋯,m,l=0,⋯,M−1) and

(32) $$\begin{array}{ccc} & & t_{1}^{0}d^{m-1}+\cdots +t_{m}^{0}=0{⊕}_{2}l_{1}{⊕}_{3}l_{2}{⊕}_{4}\cdots {⊕}_{M}l_{M-1}\\ & & t_{1}^{1}d^{m-1}+\cdots +t_{m}^{1}=1{⊕}_{2}l_{1}{⊕}_{3}l_{2}{⊕}_{4}\cdots {⊕}_{M}l_{M-1}\\ & & \cdots \\ & & t_{1}^{M-1}d^{m-1}+\cdots +t_{m}^{M-1}=\left(M-1\right){⊕}_{M}l_{M-1}. \end{array}$$

Similar to the case for the hierarchical joint remote implementation of partially unknown operations of one qudit, the n senders $Alice_{1},\cdots ,Alice_{n}$ share the information of the partially unknown operation $U_{l_{1},l_{2},\cdots ,l_{M}}\left(\varphi_{0},\varphi_{1},\cdots ,\varphi_{M-1}\right)$ to be remotely implemented. That is, $Alice_{u}$(u=1,⋯,n) knows $\varphi_{u,0},\varphi_{u,1},\cdots ,\varphi_{u,M-1}$. Here, $\sum_{u=1}^{n}\varphi_{u,j}=\varphi_{j}$(j=0,1,⋯,M−1). All the senders $Alice_{1},\cdots ,Alice_{n}$ cooperate with each other to remote implement the partially unknown operations and help the remote receiver to prepare the target state.

Similar to the case for the hierarchical joint remote implementation of partially unknown operations of one qudit, the n senders $Alice_{1},\cdots ,$$Alice_{n}$, y upper-grade agents $Bob_{1},\cdots ,Bob_{y}$ and z lower-grade agents $Charlie_{1},\cdots ,Charlie_{z}$ share m (n + y + z)-qudit entangled states. One of the upper-grade agents has the qudits $b_{1},\cdots ,b_{m}$ in the arbitrary m-qudit state. For the hierarchical joint remote implementation of partially unknown operations, the upper-grade agent first carries out m C-NOT operation on his entangled particles and particles $b_{1},\cdots ,b_{m}$, and then performs Z-basis measurements on particles $b_{1},\cdots ,b_{m}$. The n senders $Alice_{1},\cdots ,$$Alice_{n}$ first perform corresponding unitary operations on their entangled particles according to the measurement result obtained by the upper-grade agent, and then implement partially unknown operations according to their information of the partially unknown operation to be remotely implemented. The receivers are hierarchized according to their abilities to reconstruct the desired state. That is, the upper-grade agents $Bob_{1},\cdots ,Bob_{y}$ can prepare the desired state with the cooperation of one of the lower-grade agents and the lower-grade agents $Charlie_{1},\cdots ,Charlie_{z}$ need the cooperation of all the other agents to prepare the target state.

To hierarchically jointly remotely implement the partially unknown operations of m qudits, the n senders $Alice_{1},\cdots ,$$Alice_{n}$, y upper-grade agents $Bob_{1},\cdots ,Bob_{y}$ and z lower-grade agents $Charlie_{1},\cdots ,Charlie_{z}$ share m (n+y+z)-qudit entangled states.

(33) |ϕ′〉=|ϕ〉⊗m=∑j11,j12,⋯,jm1,jm2=0d−1e2πidj11j12⋯e2πidjm1jm2|j11,⋯,j11〉A11,⋯,A1n|j11,⋯,j11〉B11,⋯,B1y|j12,⋯,j12〉C11,⋯,C1z⋯|jm1,⋯,jm1〉Am1,⋯,Amn|jm1,⋯,jm1〉Bm1,⋯,Bmy|jm2,⋯,jm2〉Cm1,⋯,Cmz.

where particles $A_{1u},\cdots ,A_{mu}$ belong to the sender $Alice_{u}$(u=1,⋯,n), the upper-grade agents $Bob_{k}$(k=1,⋯,y) are in possession of particles $B_{1k},\cdots ,B_{mk}$, and the lower-grade agents $Charlie_{p}$(p=1,⋯,z) are in possession of particles $C_{1p},\cdots ,C_{mp}$.

Without loss of generality, suppose $Bob_{1}$ has the qudits $b_{1},\cdots ,b_{m}$ in the arbitrary m-qudit state [89]:

(34) $$\begin{array}{c} {|\psi \rangle }_{b_{1},\cdots ,b_{m}}=\sum_{l_{1},\cdots ,l_{m}=0}^{d-1}\alpha_{l_{1},\cdots ,l_{m}}|l_{1},\cdots ,l_{m}\rangle , \end{array}$$

where

(35) $$\begin{array}{c} \sum_{l_{1},\cdots ,l_{m}=0}^{d-1}{\left|\alpha_{l_{1},\cdots ,l_{m}}\right|}^{2}. \end{array}$$

The n senders $Alice_{1},\cdots ,Alice_{n}$ want to jointly remotely implement the partially unknown operation $U_{l_{1},l_{2},\cdots ,l_{d-1}}$ and help the remote receiver prepare the target state $|\psi^{\prime }\rangle$.

(36) $$\begin{array}{ccc} |\psi^{\prime }\rangle & = & U_{l_{1},l_{2},\cdots ,l_{M-1}}|\psi \rangle \\ & = & e^{i\varphi_{0{⊕}_{2}l_{1}{⊕}_{3}l_{2}{⊕}_{4}\cdots {⊕}_{M}l_{M-1}}}\alpha_{0,\cdots ,0}|t_{1}^{0},\cdots ,t_{m}^{0}\rangle \\ & + & e^{i\varphi_{1{⊕}_{2}l_{1}{⊕}_{3}l_{2}{⊕}_{4}\cdots {⊕}_{M}l_{M-1}}}\alpha_{0,\cdots ,1}|t_{1}^{1},\cdots ,t_{m}^{1}\rangle +\cdots \\ & + & e^{i\varphi_{\left(M-1\right){⊕}_{M}l_{M-1}}}\alpha_{d-1,\cdots ,d-1}|t_{1}^{M-1},\cdots ,t_{m}^{M-1}\rangle . \end{array}$$

The state of particles $A_{11},\cdots ,C_{mz}$, $b_{1},\cdots ,b_{m}$ can be written as:

(37) |Φ〉=|ϕ〉A11,⋯,Cmz⊗|ψ〉b1,⋯,bm=∑j11,j12,⋯,jm1,jm2,l1,⋯,lm=0d−1e2πidj11j12⋯e2πidjm1jm2αl1,⋯,lm|j11,⋯,j11〉A11,⋯,A1n|j11,⋯,j11〉B11,⋯,B1y|j12,⋯,j12〉C11,⋯,C1z⋯|jm1,⋯,jm1〉Am1,⋯,Amn|jm1,⋯,jm1〉Bm1,⋯,Bmy|jm2,⋯,jm2〉Cm1,⋯,Cmz|l1,⋯,lm〉b1,⋯,bm.

For the hierarchical joint remote implementation of the partially unknown operations, $Bob_{1}$ performs m C-NOT operations on qudit $b_{v}$ and $B_{v1}$(v=1,⋯,m) by using qudit $B_{v1}$ as the control qudit. The state of particles $A_{11},\cdots ,C_{mz}$, $b_{1},\cdots ,b_{m}$ becomes $|\Phi_{1}\rangle$ after the C-NOT operations.

(38) |Φ1〉=|ϕ〉A11,⋯,Cmz⊗|ψ〉b1,⋯,bm=∑j11,j12,⋯,jm1,jm2,l1,⋯,lm=0d−1e2πidj11j12⋯e2πidjm1jm2αl1,⋯,lm|j11,⋯,j11〉A11,⋯,A1n|j11,⋯,j11〉B11,⋯,B1y|j12,⋯,j12〉C11,⋯,C1z⋯|jm1,⋯,jm1〉Am1,⋯,Amn|jm1,⋯,jm1〉Bm1,⋯,Bmy|jm2,⋯,jm2〉Cm1,⋯,Cmz|l1⊕dj11,⋯,lm⊕djm1〉b1,⋯,bm.

After the C-NOT operations, $Bob_{1}$ performs a Z-basis measurement on particles on qudit $b_{v}$(v=1,⋯,m). The state of particles $A_{11},\cdots ,C_{mz}$ becomes $|\phi_{1}\rangle$ if the measurement results are $t_{1},\cdots ,t_{m}$$\left(t_{1},\cdots ,t_{m}=0,\cdots ,d-1\right)$.

(39) |ϕ1〉=∑j11,j12,⋯,jm1,jm2=0d−1e2πidj11j12⋯e2πidjm1jm2αt1⊕d(d−j11),⋯,tm⊕d(d−jm1)|j11,⋯,j11〉A11,⋯,A1n|j11,⋯,j11〉B11,⋯,B1y|j12,⋯,j12〉C11,⋯,C1z⋯|jm1,⋯,jm1〉Am1,⋯,Amn|jm1,⋯,jm1〉Bm1,⋯,Bmy|jm2,⋯,jm2〉Cm1,⋯,Cmz.

Similar to the case for the hierarchical joint remote implementation of partially unknown operations of one qudit, $Alice_{u}$(u=1,⋯,n) first performs a single-qudit operation $X_{t_{1}},\cdots ,X_{t_{m}}$ on particles $A_{1u},\cdots ,A_{mu}$ according to $Bob_{1}$’s measurement results $t_{1},\cdots ,t_{m}$, and then applies the partially unknown operation $U_{l_{1},l_{2},\cdots ,l_{M}}\left(\varphi_{u,0},\cdots ,\varphi_{u,M-1}\right)$ on its particles $A_{1u},\cdots ,A_{mu}$ in accordance with its knowledge of $\varphi_{u,0},\cdots ,\varphi_{u,M-1}$ and perform X-basis measurements on particles $A_{1u},\cdots ,A_{mu}$. The state of particles $A_{11},\cdots ,C_{mz}$ becomes $|\phi_{2}\rangle$ after $Alice_{u}$(u=1,⋯,n) performs single-qudit operation $X_{t_{1}},\cdots ,X_{t_{m}}$ on its particles $A_{1u},\cdots ,A_{mu}$.

(40) |ϕ2〉=∑j11,j12,⋯,jm1,jm2=0d−1e2πid(t1−j11)j12⋯e2πid(tm−jm1)jm2αj11,⋯,jm1|j11,⋯,j11〉A11,⋯,A1n|t1⊕d(d−j11),⋯,t1⊕d(d−j11)〉B11,⋯,B1y|j12,⋯,j12〉C11,⋯,C1z⋯|jm1,⋯,jm1〉Am1,⋯,Amn|tm⊕d(d−jm1),⋯,tm⊕d(d−jm1)〉Bm1,⋯,Bmy|jm2,⋯,jm2〉Cm1,⋯,Cmz.

After performing a single-qudit operation $X_{t_{1}},\cdots ,X_{t_{m}}$ on particles $A_{1u},\cdots ,A_{mu}$(u=1,⋯,n), $Alice_{u}$ applies partially unknown operation $U_{l_{1},l_{2},\cdots ,l_{M}}\left(\varphi_{u,0},\cdots ,\varphi_{u,M-1}\right)$ on its particles $A_{1u},\cdots ,$$A_{mu}$. The state of particles $A_{11},\cdots ,C_{mz}$ becomes

(41) $$\begin{array}{ccc} |\phi_{3}\rangle & = & \sum_{j_{12},\cdots ,j_{m2}=0}^{d-1}|j_{12},\cdots ,j_{12}\rangle_{C_{11},\cdots ,C_{1z}}\cdots {|j_{m2},\cdots ,j_{m2}\rangle }_{C_{m1},\cdots ,C_{mz}}\\ & & [\alpha_{0,\cdots ,0}e^{\frac{2\pi i}{d}t_{1}j_{12}}\cdots e^{\frac{2\pi i}{d}t_{m}j_{m2}}e^{i\varphi_{1,0{⊕}_{2}l_{1}\cdots {⊕}_{M}l_{M-1}}}\\ & & |t_{1}^{0},\cdots ,t_{m}^{0}\rangle_{A_{11},\cdots ,A_{m1}}\cdots e^{i\varphi_{n,0{⊕}_{2}l_{1}\cdots {⊕}_{M}l_{M-1}}}{|t_{1}^{0},\cdots ,t_{m}^{0}\rangle }_{A_{1n},\cdots ,A_{mn}}\\ & & |t_{1},\cdots ,t_{1}\rangle_{B_{11},\cdots ,B_{1y}}\cdots {|t_{m},\cdots ,t_{m}\rangle }_{B_{m1},\cdots ,B_{my}}\\ & + & \alpha_{0,\cdots ,1}e^{\frac{2\pi i}{d}t_{1}j_{12}}\cdots e^{\frac{2\pi i}{d}\left(t_{m}+d-1\right)j_{m2}}e^{i\varphi_{1,1{⊕}_{2}l_{1}\cdots {⊕}_{M}l_{M-1}}}\\ & & |t_{1}^{1},\cdots ,t_{m}^{1}\rangle_{A_{11},\cdots ,A_{m1}}\cdots e^{i\varphi_{n,1{⊕}_{2}l_{1}\cdots {⊕}_{M}l_{M-1}}}{|t_{1}^{1},\cdots ,t_{m}^{1}\rangle }_{A_{1n},\cdots ,A_{mn}}\\ & & |t_{1},\cdots ,t_{1}\rangle_{B_{11},\cdots ,B_{1y}}\cdots {|t_{m}{⊕}_{d}\left(d-1\right),\cdots ,t_{m}{⊕}_{d}\left(d-1\right)\rangle }_{B_{m1},\cdots ,B_{my}}\\ & + & \cdots \\ & + & \alpha_{d-1,\cdots ,d-1}e^{\frac{2\pi i}{d}\left(t_{1}+1\right)j_{12}}\cdots \;e^{\frac{2\pi i}{d}\left(t_{m}+1\right)j_{m2}}e^{i\varphi_{1,\left(M-1\right){⊕}_{M}l_{M-1}}}\\ & & |t_{1}^{M-1},\cdots ,t_{m}^{M-1}\rangle_{A_{11},\cdots ,A_{m1}}\cdots e^{i\varphi_{n,\left(M-1\right){⊕}_{M}l_{M-1}}}\\ & & |t_{1}^{M-1},\cdots ,t_{m}^{M-1}\rangle_{A_{1n},\cdots ,A_{mn}}{|t_{1}{⊕}_{d}1,\cdots ,t_{1}{⊕}_{d}1\rangle }_{B_{11},\cdots ,B_{1y}}\cdots \\ & & |t_{m}{⊕}_{d}1,\cdots ,t_{m}{⊕}_{d}1\rangle_{B_{m1},\cdots ,B_{my}}].\\ & = & \sum_{j_{12},\cdots ,j_{m2}=0}^{d-1}|j_{12},\cdots ,j_{12}\rangle_{C_{11},\cdots ,C_{1z}}\cdots {|j_{m2},\cdots ,j_{m2}\rangle }_{C_{m1},\cdots ,C_{mz}}\\ & & [\alpha_{0,\cdots ,0}e^{\frac{2\pi i}{d}t_{1}j_{12}}\cdots e^{\frac{2\pi i}{d}t_{m}j_{m2}}e^{i\varphi_{0{⊕}_{2}l_{1}\cdots {⊕}_{M}l_{M-1}}}\\ & & |t_{1}^{0},\cdots ,t_{m}^{0}\rangle_{A_{11},\cdots ,A_{m1}}\cdots {|t_{1}^{0},\cdots ,t_{m}^{0}\rangle }_{A_{1n},\cdots ,A_{mn}}\\ & & |t_{1},\cdots ,t_{1}\rangle_{B_{11},\cdots ,B_{1y}}\cdots {|t_{m},\cdots ,t_{m}\rangle }_{B_{m1},\cdots ,B_{my}}\\ & + & \alpha_{0,\cdots ,1}e^{\frac{2\pi i}{d}t_{1}j_{12}}\cdots e^{\frac{2\pi i}{d}\left(t_{m}+d-1\right)j_{m2}}e^{i\varphi_{1{⊕}_{2}l_{1}\cdots {⊕}_{M}l_{M-1}}}\\ & & |t_{1}^{1},\cdots ,t_{m}^{1}\rangle_{A_{11},\cdots ,A_{m1}}\cdots {|t_{1}^{1},\cdots ,t_{m}^{1}\rangle }_{A_{1n},\cdots ,A_{mn}}\\ & & |t_{1},\cdots ,t_{1}\rangle_{B_{11},\cdots ,B_{1y}}\cdots {|t_{m}{⊕}_{d}\left(d-1\right),\cdots ,t_{m}{⊕}_{d}\left(d-1\right)\rangle }_{B_{m1},\cdots ,B_{my}}\\ & + & \cdots \\ & + & \alpha_{d-1,\cdots ,d-1}e^{\frac{2\pi i}{d}\left(t_{1}+1\right)j_{12}}\cdots \;e^{\frac{2\pi i}{d}\left(t_{m}+1\right)j_{m2}}e^{i\varphi_{\left(M-1\right){⊕}_{M}l_{M-1}}}\\ & & |t_{1}^{M-1},\cdots ,t_{m}^{M-1}\rangle_{A_{11},\cdots ,A_{m1}}\cdots {|t_{1}^{M-1},\cdots ,t_{m}^{M-1}\rangle }_{A_{1n},\cdots ,A_{mn}}\\ & & |t_{1}{⊕}_{d}1,\cdots ,t_{1}{⊕}_{d}{1\rangle }_{B_{11},\cdots ,B_{1y}}\cdots |t_{m}{⊕}_{d}1,\cdots ,t_{m}{⊕}_{d}1\rangle_{B_{m1},\cdots ,B_{my}}]. \end{array}$$

To implement the partially unknown operations remotely, $Alice_{u}$ performs X-basis measurements on its particles $A_{1u},\cdots ,$$A_{mu}$. The state of particles $A_{11},\cdots ,C_{mz}$ can be rewritten as:

(42) |ϕ3〉=∑r11,⋯,rmn,j12,⋯,jm2=0d−1|j12,⋯,j12〉C11,⋯,C1z⋯|jm2,⋯,jm2〉Cm1,⋯,Cmz[α0,⋯,0e2πidt1j12⋯e2πidtmjm2eiφ0⊕2l1⋯⊕MlM−1e−2πid(r11+⋯r1n)t10⋯e−2πid(rm1+⋯rmn)tm0|r11〉x,⋯,|rm1〉x,⋯,|r1n〉x,⋯,|rmn〉x|t1,⋯,t1〉⋯|tm,⋯,tm〉+α0,⋯,1e2πidt1j12⋯e2πid(tm+d−1)jm2eiφ1⊕2l1⋯⊕MlM−1〉e−2πid(r11+⋯r1n)t11⋯e−2πid(rm1+⋯rmn)tm1|r11〉x,⋯,|rm1〉x,⋯,|r1n〉x,⋯,|rmn〉x|t1,⋯,t1〉⋯|tm⊕d(d−1),⋯,tm⊕d(d−1)〉+⋯+αd−1,⋯,d−1e2πid(t1+1)j12⋯e2πid(tm+1)jm2eiφ(M−1)⊕MlM−1e−2πid(r11+⋯r1n)t1M−1⋯e−2πid(rm1+⋯rmn)tmM−1|r11〉x,⋯,|rm1〉x,⋯,|r1n〉x,⋯,|rmn〉x|t1⊕d1〉⊗⋯⊗|t1⊕d1〉⋯|tm⊕d1〉⊗⋯⊗|tm⊕d1〉]A11⋯Am1⋯A1n⋯AmnB11⋯B1y⋯Bm1⋯Bmy.

The state of particles $B_{11},\cdots ,C_{mz}$ becomes $|\phi_{4}\rangle$ if the measurement results obtained by $Alice_{u}$(u=1,⋯,n) are $|r_{1u}\rangle_{x},\cdots ,{|r_{mu}\rangle }_{x}$.

(43) $$\begin{array}{ccc} |\phi_{4}\rangle & = & \sum_{j_{12},\cdots ,j_{m2}=0}^{d-1}|j_{12},\cdots ,j_{12}\rangle_{C_{11},\cdots ,C_{1z}}\cdots {|j_{m2},\cdots ,j_{m2}\rangle }_{C_{m1},\cdots ,C_{mz}}\\ & & [\alpha_{0,\cdots ,0}e^{\frac{2\pi i}{d}t_{1}j_{12}}\cdots e^{\frac{2\pi i}{d}t_{m}j_{m2}}e^{i\varphi_{0{⊕}_{2}l_{1}\cdots {⊕}_{M}l_{M-1}}}\\ & & e^{-\frac{2\pi i}{d}r_{1}t_{1}^{0}}\cdots e^{-\frac{2\pi i}{d}r_{m}t_{m}^{0}}|t_{1},\cdots ,t_{1}\rangle \cdots |t_{m},\cdots ,t_{m}\rangle \\ & + & \alpha_{0,\cdots ,1}e^{\frac{2\pi i}{d}t_{1}j_{12}}\cdots e^{\frac{2\pi i}{d}\left(t_{m}+d-1\right)j_{m2}}e^{i\varphi_{1{⊕}_{2}l_{1}\cdots {⊕}_{M}l_{M-1}}}\\ & & e^{-\frac{2\pi i}{d}r_{1}t_{1}^{1}}\cdots e^{-\frac{2\pi i}{d}r_{m}t_{m}^{1}}|t_{1},\cdots ,t_{1}\rangle \\ & & \cdots |t_{m}{⊕}_{d}\left(d-1\right),\cdots ,t_{m}{⊕}_{d}\left(d-1\right)\rangle +\cdots \\ & + & \alpha_{d-1,\cdots ,d-1}e^{\frac{2\pi i}{d}\left(t_{1}+1\right)j_{12}}\cdots \;e^{\frac{2\pi i}{d}\left(t_{m}+1\right)j_{m2}}\\ & & e^{i\varphi_{\left(M-1\right){⊕}_{M}l_{M-1}}}e^{-\frac{2\pi i}{d}r_{1}t_{1}^{M-1}}\cdots e^{-\frac{2\pi i}{d}r_{m}t_{m}^{M-1}}\\ & & |t_{1}{⊕}_{d}1\rangle ⊗\cdots ⊗|t_{1}{⊕}_{d}1\rangle \cdots \\ & & |t_{m}{⊕}_{d}1\rangle ⊗\cdots ⊗|t_{m}{⊕}_{d}1{\rangle ]}_{B_{11}\cdots B_{1y}\cdots B_{m1}\cdots B_{my}}, \end{array}$$

where $r_{v}=r_{v1}+\cdots r_{vn}$(v=1,⋯,m).

**Figure 3:**
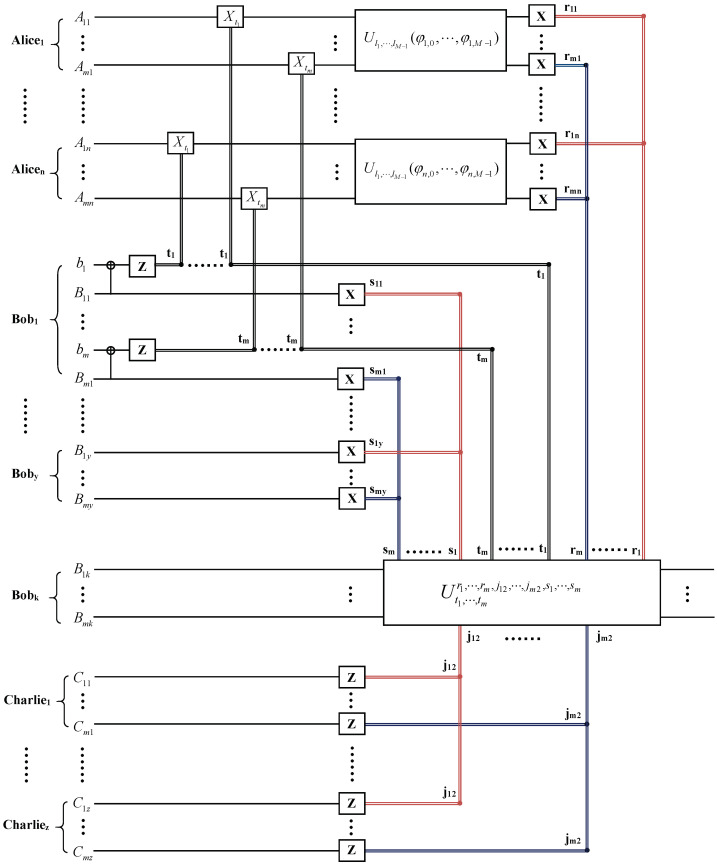
The quantum circuit for the hierarchial joint remote implementation of partially unknown operations of m qudits with upper-grade agent $Bob_{k}$. $U_{t_{1},\cdots ,t_{m}}^{r_{1},\cdots ,r_{m},j_{12},\cdots ,j_{m2},s_{1},\cdots ,s_{m}}$ in the solid-line box denotes the unitary operation performed by the receiver.

Firstly, we suppose that the agents agree to reconstruct the desired state $|\psi^{\prime }\rangle$ at the upper-grade agent $Bob_{k}$’s (k=1,⋯,y) site. The quantum circuit for the hierarchial joint remote implementation of partially unknown operations of m qudits with upper-grade agent is shown in Figure 3. To reconstruct the desired state at $Bob_{k}$’s site, the lower-grade agent $Charlie_{p}$(p=1,⋯,z) performs Z-basis measurement on his qudits $C_{1p},\cdots ,C_{mp}$. The other upper-grade agents $Bob_{1},\cdots ,Bob_{k-1},Bob_{k+1},\cdots ,Bob_{y}$ perform X-basis measurements on their qudits $B_{11},\cdots ,B_{m1},\cdots ,Bob_{1,k-1},\cdots ,Bob_{m,k-1},Bob_{1,k+1},\cdots ,$$Bob_{m,k+1},\cdots ,Bob_{1,y},\cdots$, $Bob_{m,y}$. The receiver $Bob_{k}$ can reconstruct the desired state by performing unitary operation on his qudits $Bob_{1,k},\cdots ,Bob_{m,k}$ according to other upper-grade agents’ measurement results and one of the lower-grade agents’ measurements.

The state of particles $B_{11},\cdots ,B_{1y},\cdots ,B_{m1},\cdots ,B_{my}$ becomes $|\phi_{5}\rangle$ if the measurement results obtained by $Charlie_{p}$ are $|j_{12}\rangle_{C_{1p}},\cdots ,{|j_{m2}\rangle }_{C_{mp}}$.

(44) $$\begin{array}{ccc} |\phi_{5}\rangle & = & [\alpha_{0,\cdots ,0}e^{\frac{2\pi i}{d}t_{1}j_{12}}\cdots e^{\frac{2\pi i}{d}t_{m}j_{m2}}e^{i\varphi_{0{⊕}_{2}l_{1}\cdots {⊕}_{M}l_{M-1}}}\\ & & e^{-\frac{2\pi i}{d}r_{1}t_{1}^{0}}\cdots e^{-\frac{2\pi i}{d}r_{m}t_{m}^{0}}|t_{1},\cdots ,t_{1}\rangle \cdots |t_{m},\cdots ,t_{m}\rangle \\ & + & \alpha_{0,\cdots ,1}e^{\frac{2\pi i}{d}t_{1}j_{12}}\cdots e^{\frac{2\pi i}{d}\left(t_{m}+d-1\right)j_{m2}}e^{i\varphi_{1{⊕}_{2}l_{1}\cdots {⊕}_{M}l_{M-1}}}\\ & & e^{-\frac{2\pi i}{d}r_{1}t_{1}^{1}}\cdots e^{-\frac{2\pi i}{d}r_{m}t_{m}^{1}}|t_{1},\cdots ,t_{1}\rangle \\ & & \cdots |t_{m}{⊕}_{d}\left(d-1\right),\cdots ,t_{m}{⊕}_{d}\left(d-1\right)\rangle +\cdots \\ & + & \alpha_{d-1,\cdots ,d-1}e^{\frac{2\pi i}{d}\left(t_{1}+1\right)j_{12}}\cdots \;e^{\frac{2\pi i}{d}\left(t_{m}+1\right)j_{m2}}\\ & & e^{i\varphi_{\left(M-1\right){⊕}_{M}l_{M-1}}}e^{-\frac{2\pi i}{d}r_{1}t_{1}^{M-1}}\cdots e^{-\frac{2\pi i}{d}r_{m}t_{m}^{M-1}}\\ & & |t_{1}{⊕}_{d}1\rangle ⊗\cdots ⊗|t_{1}{⊕}_{d}1\rangle \cdots \\ & & |t_{m}{⊕}_{d}1\rangle ⊗\cdots ⊗|t_{m}{⊕}_{d}1{\rangle ]}_{B_{11}\cdots B_{1y}\cdots B_{m1}\cdots B_{my}}. \end{array}$$

To hierarchically jointly remotely implement the partially unknown operations, the other upper-grade agents $Bob_{1},\cdots ,Bob_{k-1},Bob_{k+1},\cdots ,Bob_{y}$ perform X-basis measurements on their qudits $B_{11},\cdots ,B_{m1},\cdots ,B_{1,k-1},\cdots ,B_{m,k-1},B_{1,k+1},\cdots ,$$B_{m,k+1},\cdots ,B_{1,y},\cdots ,$$Bob_{m,y}$. The state of particles $B_{11},\cdots ,B_{1y},\cdots ,B_{m1},\cdots ,B_{my}$ can be rewritten as:

(45) |ϕ5〉=∑s11,⋯,s1y,⋯,sm1,⋯,smy=0d−1[α0,⋯,0e2πidt1j12⋯e2πidtmjm2eiφ0⊕2l1⋯⊕MlM−1e−2πidr1t10⋯e−2πidrmtm0e−2πids1t1⋯e−2πidsmtm|s11〉x⋯|s1,k−1〉x|t1〉|s1,k+1〉x⋯|s1,y〉x⋯|sm1〉x⋯|sm,k−1〉x|tm〉|sm,k+1〉x⋯|sm,y〉x+α0,⋯,1e2πidt1j12⋯e2πid(tm+d−1)jm2eiφ1⊕2l1⋯⊕MlM−1e−2πidr1t11⋯e−2πidrmtm1e−2πids1t1⋯e−2πidsm(tm+d−1)|s11〉x⋯|s1,k−1〉x|t1〉|s1,k+1〉x⋯|s1,y〉x⋯|sm1〉x⋯|sm,k−1〉x|tm⊕d(d−1)〉|sm,k+1〉x⋯|sm,y〉x+⋯+αd−1,⋯,d−1e2πid(t1+1)j12⋯e2πid(tm+1)jm2eiφ(M−1)⊕MlM−1e−2πidr1t1M−1⋯e−2πidrmtmM−1e−2πids1(t1+1)⋯e−2πidsm(tm+1)|s11〉x⋯|s1,k−1〉x|t1⊕d1〉|s1,k+1〉x⋯|s1,y〉x⋯|sm1〉x⋯|sm,k−1〉x|tm⊕d1〉|sm,k+1〉x⋯|sm,y〉x]B11⋯B1y⋯Bm1⋯Bmy,

where $s_{v}=s_{v1}+\cdots +s_{v,k-1}+s_{v,k+1}+\cdots +s_{vy}$(v=1,⋯,m).

The state of particles $B_{1k},\cdots ,B_{mk}$ becomes $|\phi_{6}\rangle$ if the measurement results obtained by $Bob_{1},\cdots ,Bob_{k-1},Bob_{k+1},\cdots ,Bob_{y}$ are $s_{11},\cdots ,s_{m1}$, ⋯, $s_{1,k-1},\cdots ,s_{m,k-1}$, ⋯, $s_{1,k+1},\cdots ,$$s_{m,k+1}$, ⋯, $s_{1y},\cdots ,s_{my}$.

(46) $$\begin{array}{ccc} |\phi_{6}\rangle & = & [\alpha_{0,\cdots ,0}e^{\frac{2\pi i}{d}t_{1}j_{12}}\cdots e^{\frac{2\pi i}{d}t_{m}j_{m2}}e^{i\varphi_{0{⊕}_{2}l_{1}\cdots {⊕}_{M}l_{M-1}}}\\ & & e^{-\frac{2\pi i}{d}r_{1}t_{1}^{0}}\cdots e^{-\frac{2\pi i}{d}r_{m}t_{m}^{0}}e^{-\frac{2\pi i}{d}s_{1}t_{1}}\cdots e^{-\frac{2\pi i}{d}s_{m}t_{m}}|t_{1}\rangle \cdots |t_{m}\rangle \\ & + & \alpha_{0,\cdots ,1}e^{\frac{2\pi i}{d}t_{1}j_{12}}\cdots e^{\frac{2\pi i}{d}\left(t_{m}+d-1\right)j_{m2}}e^{i\varphi_{1{⊕}_{2}l_{1}\cdots {⊕}_{M}l_{M-1}}}\\ & & e^{-\frac{2\pi i}{d}r_{1}t_{1}^{1}}\cdots e^{-\frac{2\pi i}{d}r_{m}t_{m}^{1}}e^{-\frac{2\pi i}{d}s_{1}t_{1}}\cdots e^{-\frac{2\pi i}{d}s_{m}\left(t_{m}+d-1\right)}\\ & & |t_{1}\rangle \cdots |t_{m}{⊕}_{d}\left(d-1\right)\rangle +\cdots \\ & + & \alpha_{d-1,\cdots ,d-1}e^{\frac{2\pi i}{d}\left(t_{1}+1\right)j_{12}}\cdots \;e^{\frac{2\pi i}{d}\left(t_{m}+1\right)j_{m2}}e^{i\varphi_{\left(M-1\right){⊕}_{M}l_{M-1}}}\\ & & e^{-\frac{2\pi i}{d}r_{1}t_{1}^{M-1}}\cdots e^{-\frac{2\pi i}{d}r_{m}t_{m}^{M-1}}e^{-\frac{2\pi i}{d}s_{1}\left(t_{1}+1\right)}\cdots e^{-\frac{2\pi i}{d}s_{m}\left(t_{m}+1\right)}\\ & & |t_{1}{⊕}_{d}1\rangle \cdots |t_{m}{⊕}_{d}1{\rangle ]}_{B_{1k}\cdots B_{mk}}. \end{array}$$

Similar to the case for remote implementation of partially unknown operations of one qudit, the m-qudit operation

(47) $$\begin{array}{ccc} & & U_{t_{1},\cdots ,t_{m}}^{r_{1},\cdots ,r_{m},j_{12},\cdots ,j_{m2},s_{1},\cdots ,s_{m}}\\ & = & e^{\frac{2\pi i}{d}t_{1}\left(s_{1}-j_{12}\right)}\cdots e^{\frac{2\pi i}{d}t_{m}\left(s_{m}-j_{m2}\right)}e^{\frac{2\pi i}{d}r_{1}t_{1}^{0}}\cdots e^{-\frac{2\pi i}{d}r_{m}t_{m}^{0}}\\ & & |t_{1}^{0},\cdots ,t_{m}^{0}\rangle \langle t_{1},\cdots ,t_{m}|\\ & + & e^{\frac{2\pi i}{d}t_{1}\left(s_{1}-j_{12}\right)}\cdots e^{\frac{2\pi i}{d}\left(t_{m}+d-1\right)\left(s_{m}-j_{m2}\right)}e^{\frac{2\pi i}{d}r_{1}t_{1}^{1}}\cdots e^{\frac{2\pi i}{d}r_{m}t_{m}^{1}}\\ & & |t_{1}^{1},\cdots ,t_{m}^{1}\rangle \langle t_{1},\cdots ,t_{m}{⊕}_{d}\left(d-1\right)|+\cdots \\ & + & e^{\frac{2\pi i}{d}\left(t_{1}+1\right)\left(s_{1}-j_{12}\right)}\cdots \;e^{\frac{2\pi i}{d}\left(t_{m}+1\right)\left(s_{m}-j_{m2}\right)}e^{\frac{2\pi i}{d}r_{1}t_{1}^{M-1}}\cdots e^{\frac{2\pi i}{d}r_{m}t_{m}^{M-1}}\\ & & |t_{1}^{M-1},\cdots ,t_{m}^{M-1}\rangle \langle t_{1}{⊕}_{d}1,\cdots ,t_{m}{⊕}_{d}1|, \end{array}$$

in accordance with $Bob_{1}$’s measurement results $t_{1},\cdots ,t_{m}$, the senders’ measurement results $r_{1},\cdots ,r_{m}$, the lower-grade agents’ measurement results $j_{12},\cdots ,j_{m2}$ and the upper-grade agents’ measurement results $s_{1},\cdots ,s_{m}$ can reconstruct the desired state at $Bob_{k}$’s site.

(48) $$\begin{array}{c} {\left(U_{t_{1},\cdots ,t_{m}}^{r_{1},\cdots ,r_{m},j_{12},\cdots ,j_{m2},s_{1},\cdots ,s_{m}}\right)}_{B_{1k},\cdots ,B_{mk}}|\phi_{6}\rangle =|\psi^{\prime }\rangle . \end{array}$$

**Figure 4:**
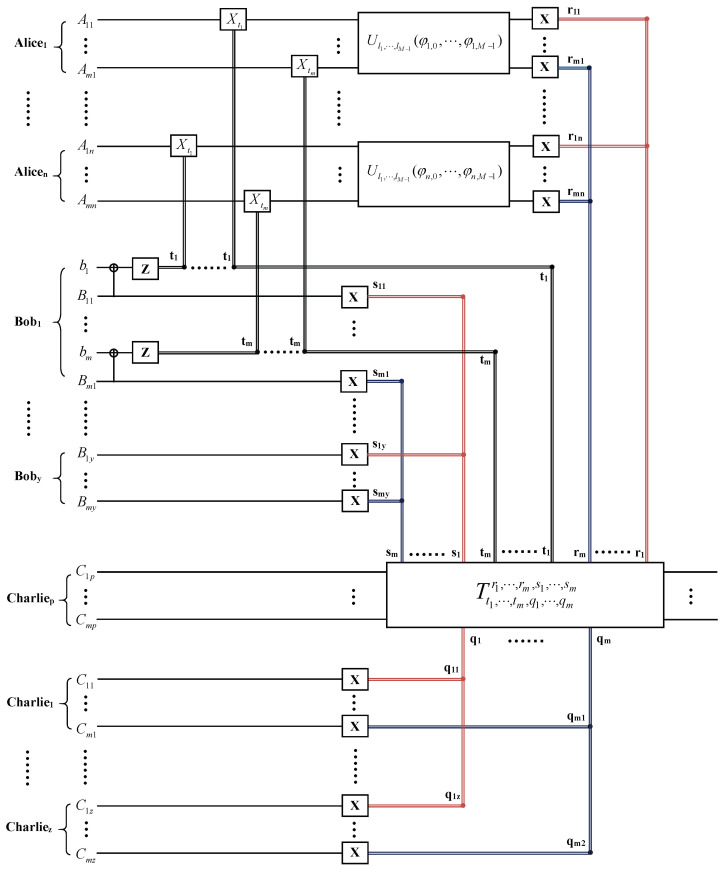
The quantum circuit for the hierarchial joint remote implementation of partially unknown operations of m qudits with lower-grade agent $Charlie_{p}$.

Now, we discuss another case in which the agents agree to reconstruct the desired state $|\psi^{\prime }\rangle$ at $Charlie_{p}$’s site (p=1,⋯,z). The quantum circuit for the hierarchial joint remote implementation of partially unknown operations of m qudits with lower-grade agent is shown in Figure 4. To reconstruct the desired state at the lower-grade agent $Charlie_{p}$’s site, the other lower-grade agents $Charlie_{1},\cdots ,Charlie_{p-1},Charlie_{p+1},\cdots ,Charlie_{z}$ and the upper-grade agents $Bob_{1},\cdots ,Bob_{y}$ perform x-basis measurements on their entangled particles $C_{11},\cdots ,C_{m1}$,⋯,$C_{p-1,1},\cdots ,C_{p-1,m}$,$C_{p+1,1},\cdots ,$$C_{p+1,m}$, $C_{1z},\cdots ,C_{mz}$ and $B_{1},\cdots ,B_{y}$, after $Alice_{u}$(l=u,⋯,n) implements partially unknown operation $U_{l_{1},l_{2},\cdots ,l_{d-1}}(\varphi_{l,0},\varphi_{l,1},\cdots$, $\varphi_{l,d-1})$ and performs x-basis measurements on its qudit $A_{u}$. The state $|\phi_{4}\rangle$ can be rewritten as:

(49) |ϕ4〉=∑j12,⋯,jm2,s11,⋯,smy,q11,⋯,qmz=0d−1e−2πidj12q1⋯e−2πidjm2qm|q11〉x,⋯,|q1,p−1〉x|j12〉|q1,p+1〉x⋯|q1,z〉x⋯|qm1〉x,⋯,|qm,p−1〉x|jm2〉|qm,p+1〉x⋯|qm,z〉x[α0,⋯,0e2πidt1j12⋯e2πidtmjm2eiφ0⊕2l1⋯⊕MlM−1e−2πidr1t10⋯e−2πidrmtm0e−2πids1t1⋯e−2πidsmtm+α0,⋯,1e2πidt1j12⋯e2πid(tm+d−1)jm2eiφ1⊕2l1⋯⊕MlM−1e−2πidr1t11⋯e−2πidrmtm1e−2πids1t1⋯e−2πidsm(tm+d−1)+⋯+αd−1,⋯,d−1e2πid(t1+1)j12⋯e2πid(tm+1)jm2eiφ(M−1)⊕MlM−1e−2πidr1t1M−1⋯e−2πidrmtmM−1e−2πids1(t1+1)⋯e−2πidsm(tm+1)],

where

(50) $$\begin{array}{ccc} q_{v} & = & q_{v,1}+\cdots +q_{v,p-1}+q_{v,p+1}+\cdots +q_{v,z},\\ s_{v} & = & s_{v,1}+\cdots +s_{v,y}. \end{array}$$

The state of particles $C_{1p},\cdots ,C_{mp}$ becomes |ω〉 if the measurement results are obtained by $Charlie_{1},\cdots ,$$Charlie_{p-1}$, $Charlie_{p+1},\cdots ,$$Charlie_{z}$ are $q_{11},\cdots ,$$q_{m1}$, ⋯, $q_{1,p-1},$$\cdots ,q_{m,p-1}$,$q_{1,p+1},\cdots ,q_{m,p+1}$, ⋯,$q_{1z},$$\cdots ,q_{mz}$.

(51) $$\begin{array}{ccc} |\omega \rangle & = & \alpha_{0,\cdots ,0}e^{i\varphi_{0{⊕}_{2}l_{1}\cdots {⊕}_{M}l_{M-1}}}e^{-\frac{2\pi i}{d}r_{1}t_{1}^{0}}\cdots e^{-\frac{2\pi i}{d}r_{m}t_{m}^{0}}\\ & & e^{-\frac{2\pi i}{d}s_{1}t_{1}}\cdots e^{-\frac{2\pi i}{d}s_{m}t_{m}}[(\sum_{j_{12}=0}^{d-1}e^{\frac{2\pi i}{d}j_{12}\left(t_{1}-q_{1}\right)}\\ & & |j_{12}\rangle )\cdots (\sum_{j_{m2}=0}^{d-1}e^{\frac{2\pi i}{d}j_{m2}\left(t_{m}-q_{m}\right)}|j_{m2}\rangle )]\\ & + & \alpha_{0,\cdots ,1}e^{i\varphi_{1{⊕}_{2}l_{1}\cdots {⊕}_{M}l_{M-1}}}e^{-\frac{2\pi i}{d}r_{1}t_{1}^{1}}\cdots e^{-\frac{2\pi i}{d}r_{m}t_{m}^{1}}\\ & & e^{-\frac{2\pi i}{d}s_{1}t_{1}}\cdots e^{-\frac{2\pi i}{d}s_{m}\left(t_{m}+d-1\right)}[(\sum_{j_{12}=0}^{d-1}e^{\frac{2\pi i}{d}j_{12}\left(t_{1}-q_{1}\right)}\\ & & |j_{12}\rangle )\cdots (\sum_{j_{m2}=0}^{d-1}e^{\frac{2\pi i}{d}j_{m2}\left(t_{m}+d-1-q_{m}\right)}|j_{m2}\rangle )]+\cdots \\ & + & \alpha_{d-1,\cdots ,d-1}e^{i\varphi_{\left(M-1\right){⊕}_{M}l_{M-1}}}e^{-\frac{2\pi i}{d}r_{1}t_{1}^{M-1}}\cdots \\ & & e^{-\frac{2\pi i}{d}r_{m}t_{m}^{M-1}}e^{-\frac{2\pi i}{d}s_{1}\left(t_{1}+1\right)}\cdots e^{-\frac{2\pi i}{d}s_{m}\left(t_{m}+1\right)}\\ & & [(\sum_{j_{12}=0}^{d-1}e^{\frac{2\pi i}{d}\left(t_{1}+1-q_{1}\right)j_{12}}|j_{12}\rangle )\cdots \\ & & (\sum_{j_{m2}=0}^{d-1}e^{\frac{2\pi i}{d}\left(t_{m}+1-q_{m}\right)j_{m2}}|j_{m2}\rangle )]. \end{array}$$

To reconstruct the desired state, $Charlie_{p}$ first performs the inverse Hadamard operations on its qudits $C_{1p},\cdots ,C_{mp}$ and then performs corresponding unitary operation on its qudits according to all the other agents’ measurement results. After $Charlie_{p}$ performs m inverse Hadamard operations on its qudits $C_{1p},\cdots ,C_{mp}$, the state of particles $C_{1p},\cdots ,C_{mp}$ becomes:

(52) $$\begin{array}{ccc} |\omega_{1}\rangle & = & \alpha_{0,\cdots ,0}e^{i\varphi_{0{⊕}_{2}l_{1}\cdots {⊕}_{M}l_{M-1}}}e^{-\frac{2\pi i}{d}r_{1}t_{1}^{0}}\cdots e^{-\frac{2\pi i}{d}r_{m}t_{m}^{0}}\\ & & e^{-\frac{2\pi i}{d}s_{1}t_{1}}\cdots e^{-\frac{2\pi i}{d}s_{m}t_{m}}[|t_{1}{⊕}_{d}\left(d-q_{1}\right)\rangle \\ & & \cdots |t_{m}{⊕}_{d}\left(d-q_{m}\right){\rangle ]}_{C_{1p},\cdots ,C_{mp}}\\ & + & \alpha_{0,\cdots ,1}e^{i\varphi_{1{⊕}_{2}l_{1}\cdots {⊕}_{M}l_{M-1}}}e^{-\frac{2\pi i}{d}r_{1}t_{1}^{1}}\cdots e^{-\frac{2\pi i}{d}r_{m}t_{m}^{1}}\\ & & e^{-\frac{2\pi i}{d}s_{1}t_{1}}\cdots e^{-\frac{2\pi i}{d}s_{m}\left(t_{m}+d-1\right)}[|t_{1}{⊕}_{d}\left(d-q_{1}\right)\rangle \\ & & \cdots |t_{m}{⊕}_{d}\left(d-1-q_{m}\right){\rangle ]}_{C_{1p},\cdots ,C_{mp}}+\cdots \\ & + & \alpha_{d-1,\cdots ,d-1}e^{i\varphi_{\left(M-1\right){⊕}_{M}l_{M-1}}}e^{-\frac{2\pi i}{d}r_{1}t_{1}^{M-1}}\cdots \\ & & e^{-\frac{2\pi i}{d}r_{m}t_{m}^{M-1}}e^{-\frac{2\pi i}{d}s_{1}\left(t_{1}+1\right)}\cdots e^{-\frac{2\pi i}{d}s_{m}\left(t_{m}+1\right)}\\ & & [|t_{1}{⊕}_{d}\left(1-q_{1}\right)\rangle \cdots |t_{m}{⊕}_{d}\left(d-q_{m}\right){\rangle ]}_{C_{1p},\cdots ,C_{mp}}. \end{array}$$

The m-qudit unitary operation

(53) $$\begin{array}{ccc} & & T_{t_{1},\cdots ,t_{m},q_{1},\cdots ,q_{m}}^{r_{1},\cdots ,r_{m},s_{1},\cdots ,s_{m}}\\ & = & e^{\frac{2\pi i}{d}r_{1}t_{1}^{0}}\cdots e^{\frac{2\pi i}{d}r_{m}t_{m}^{0}}e^{\frac{2\pi i}{d}s_{1}t_{1}}\cdots e^{\frac{2\pi i}{d}s_{m}t_{m}}\\ & & |t_{1}^{0},\cdots ,t_{m}^{0}\rangle \langle t_{1}{⊕}_{d}\left(d-q_{1}\right),\cdots ,t_{m}{⊕}_{d}\left(d-q_{m}\right)|\\ & + & e^{\frac{2\pi i}{d}r_{1}t_{1}^{1}}\cdots e^{\frac{2\pi i}{d}r_{m}t_{m}^{1}}e^{\frac{2\pi i}{d}s_{1}t_{1}}\cdots e^{\frac{2\pi i}{d}s_{m}\left(t_{m}+d-1\right)}\\ & & |t_{1}^{1},\cdots ,t_{m}^{1}\rangle \langle t_{1}{⊕}_{d}\left(d-q_{1}\right),\cdots ,t_{m}{⊕}_{d}\left(d-1-q_{m}\right)|\\ & & +\cdots \\ & + & e^{\frac{2\pi i}{d}r_{1}t_{1}^{M-1}}\cdots e^{\frac{2\pi i}{d}r_{m}t_{m}^{M-1}}e^{\frac{2\pi i}{d}s_{1}\left(t_{1}+1\right)}\cdots e^{\frac{2\pi i}{d}s_{m}\left(t_{m}+1\right)}\\ & & |t_{1}^{M-1},\cdots ,t_{m}^{M-1}\rangle \langle t_{1}{⊕}_{d}\left(1-q_{1}\right),\cdots ,t_{m}{⊕}_{d}\left(d-q_{m}\right)|, \end{array}$$

in accordance with the measurement results $t_{1},\cdots ,t_{m},$$q_{1},\cdots ,q_{m},r_{1},\cdots ,r_{m},s_{1},\cdots ,s_{m}$ obtained by the other agents can reconstruct the desired state $|\psi^{\prime }\rangle$ at $Charlie_{p}$’s site.

(54) $$\begin{array}{ccc} & & T_{t_{1},\cdots ,t_{m},q_{1},\cdots ,q_{m}}^{r_{1},\cdots ,r_{m},s_{1},\cdots ,s_{m}}|\omega_{1}\rangle_{C_{1p},\cdots ,C_{mp}}=|\psi^{\prime }\rangle . \end{array}$$

Similar to the case for remotely implementing partially unknown operations of one qudit, the protocol for the remote implementation of partially unknown operations of m qudits has the advantage of possessing a channel capacity by transmitting $d^{m}$ coefficients $\varphi_{0},\cdots ,\varphi_{d^{m}-1}$ via m multiparticle entangled states. The protocol is more convenient in application, since partially unknown operations of m qudits can be remotely implemented with less resources than that in bidirectional teleportation. Similar to the case for remotely implementing partially unknown operations of m qubits, the protocol for the remote implementation of partially unknown operations of m qudits plays an important role in distributed quantum computation, since the partially unknown operations of m qudits are not reducible to the direct products of partially unknown operations of one qudit. Since high-dimensional multiphotonic operations have been experimentally realized with an ancilla state and quantum nondemolition measurements, and a high-dimensional multiqudit state has been demonstrated via photon’s frequency degree of freedom, the protocol for the remote implementation of partially unknown operations of m qudits can be realized with current techniques [90].

## 4. Discussion and Summary

In Ref. [58],the two agents Alice and Bob can exploit the nonlocality of two-qubit entangled state to avoid the requirement that the receivers are hierarchized in accordance with their abilities to reconstruct the desired state in controlled remote implementation of the partially unknown operations of one qubit with multiparticle entangled state. However, when the protocol becomes a hierarchical controlled joint remote implementation of partially unknown operations of m qudits, the approach in Ref. [58]. does not work. In contrast to Ref. [48], the information of the partially unknown operations to be remotely implemented is shared by the n senders and the receiver cannot reconstruct the desired state if it does not cooperate with all the senders. This result will enhance the security of quantum operation remote implementation in long-distance quantum communication.

In summary, we propose a scheme for the hierarchical joint remote implementation of partially unknown operations of m qudits belonging to restricted sets by using m multiparticle entangled states as the quantum channel. The n senders share the information of the partially unknown operations to be remotely implemented and perform quantum operations on their entangled particles according to their knowledge of the quantum operation to be remotely implemented. The lower-grade agents perform z-basis measurements if the agents agree to reconstruct the desired state at the upper-grade agent’s site. The upper-grade agent needs only to cooperate with one of the lower-grade agents to reconstruct the desired state. The other agents perform x-basis measurements if the agents agree to reconstruct the desired state at the lower-grade agent’s site. The lower-grade agent needs all the other agents’ cooperation to reconstruct the desired state. This protocol has the advantage of having high channel capacity in long-distance quantum communication by using high-dimensional quantum entangled states as the quantum channel for joint implementing the partially unknown operations of m qudits.

## Data Availability

Data is contained within the article.
